# Author Correction: Divergent single cell transcriptome and epigenome alterations in ALS and FTD patients with C9orf72 mutation

**DOI:** 10.1038/s41467-024-53972-1

**Published:** 2024-11-06

**Authors:** Junhao Li, Manoj K. Jaiswal, Jo-Fan Chien, Alexey Kozlenkov, Jinyoung Jung, Ping Zhou, Mahammad Gardashli, Luc J. Pregent, Erica Engelberg-Cook, Dennis W. Dickson, Veronique V. Belzil, Eran A. Mukamel, Stella Dracheva

**Affiliations:** 1 Department of Cognitive Science, University of California San Diego, La Jolla, CA 92037 US; 2 Friedman Brain Institute and Department of Psychiatry, Icahn School of Medicine at Mount Sinai, New York, NY 10029 US; 3 Department of Physics, University of California San Diego, La Jolla, CA 92037 US; 4 Department of Neuroscience, Mayo Clinic, Jacksonville, FL 32224 US; 5 Research & Development and VISN2 MIREC, James J, Peters VA Medical Center, Bronx, NY 10468 US

Correction to: *Nature Communications* 10.1038/s41467-023-41033-y, published online 15 September 2023

The original version of this Article contained blurry figures in the Supplementary information file. In the original Supplementary information file, Supplementary Figs. 8, 10 and 11 were inadvertently of lower resolution, making them blurry and illegible.

The correct version of the Supplementary information file is:

Revised Supplementary Information

Which replaces the previous incorrect version:

Original Supplementary Information

The Supplementary information file has been corrected in both the PDF and HTML versions of the Article.

## Supplementary information

Original Supplementary Information

Revised Supplementary Information

