# Supplementary material for: Divergent single cell transcriptome and epigenome alterations in ALS and FTD patients with C9orf72 mutation
Source: Nat Commun. 2023 Sep 15;14:5714. doi: 10.1038/s41467-023-41033-y (PMC10504300; doi:10.1038/s41467-023-41033-y)
Supplement: Supplementary file 1 — Supplementary Information [file 41467_2023_41033_MOESM1_ESM.pdf]

Supplementary Information

Supplementary Figures

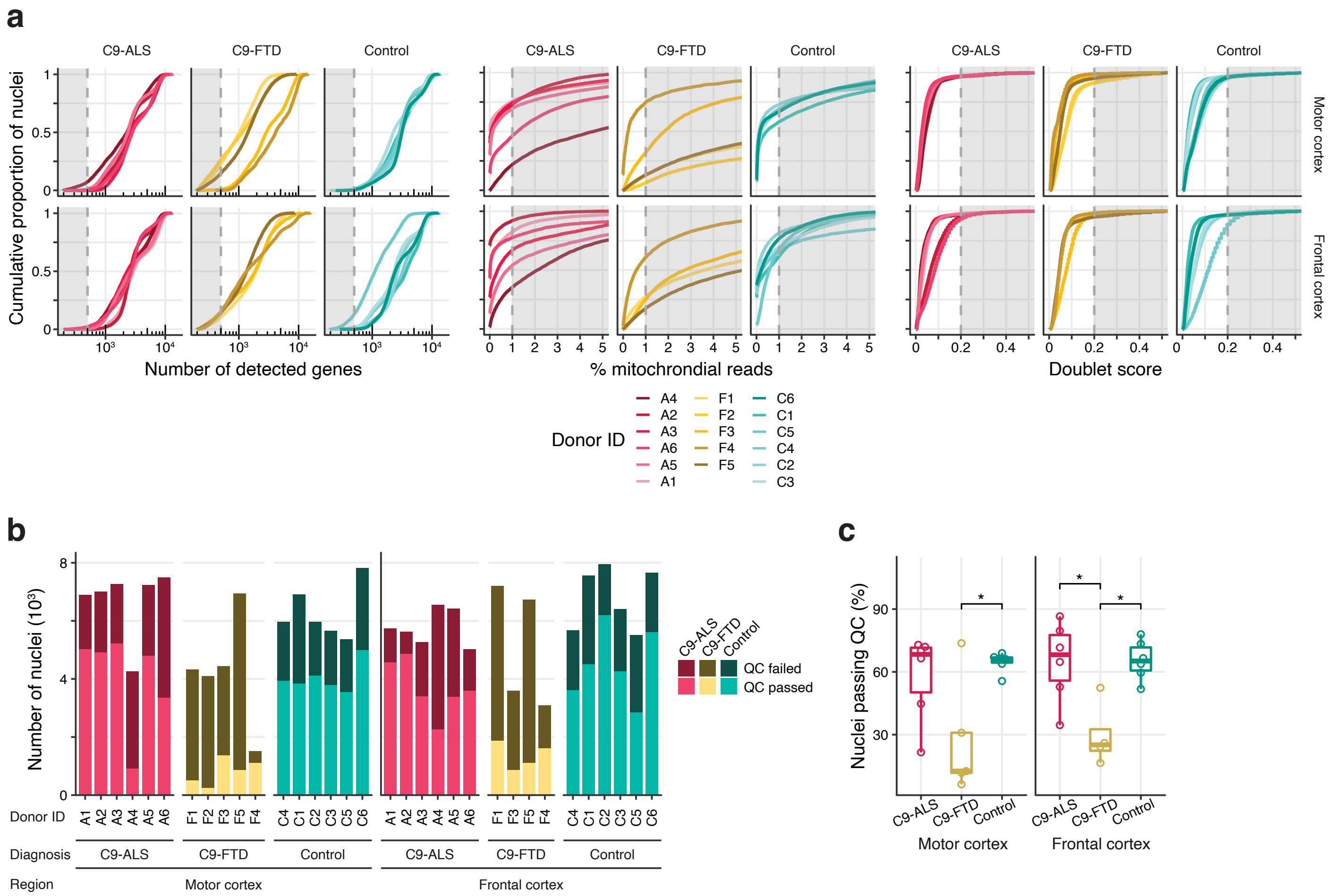

Supplementary Fig. 1. Quality control (QC) metrics for snRNA-seq data. (a) Cumulative proportion of the number of detected genes, percentage of reads mapped to the mitochondrial genome, and doublet score (from scrublet[1]) for nuclei in each snRNA-seq sample, grouped by donor ID, diagnosis and brain region. Shaded areas in gray mark the nuclei that failed the QC filtering (number of detected genes > 500, % mitochondrial reads < 1, or doublet score < 0.2). Nuclei in the C9-FTD samples failed QC mainly due to high percentage of mitochondrial reads. (b) Summary of the total number of collected nuclei, and the number that passed QC, in each sample. (c) Proportion of nuclei that passed QC. Circles represent biologically independent individual donors; N = 6 C9-ALS and 6 control samples for both brain regions, and N = 5 and 4 C9-FTD samples in motor cortex and frontal cortex, respectively. In each box plot, the lower and upper hinges correspond to the first and third quartiles; the whiskers extend 1.5 * IQR (interquartile range) away from the hinges; and the center denotes the median. *, two-sided Welch's t-test p = 0.038, 0.014, and 0.012 for the comparisons from left to right, respectively. Source data are provided as a Source Data file.

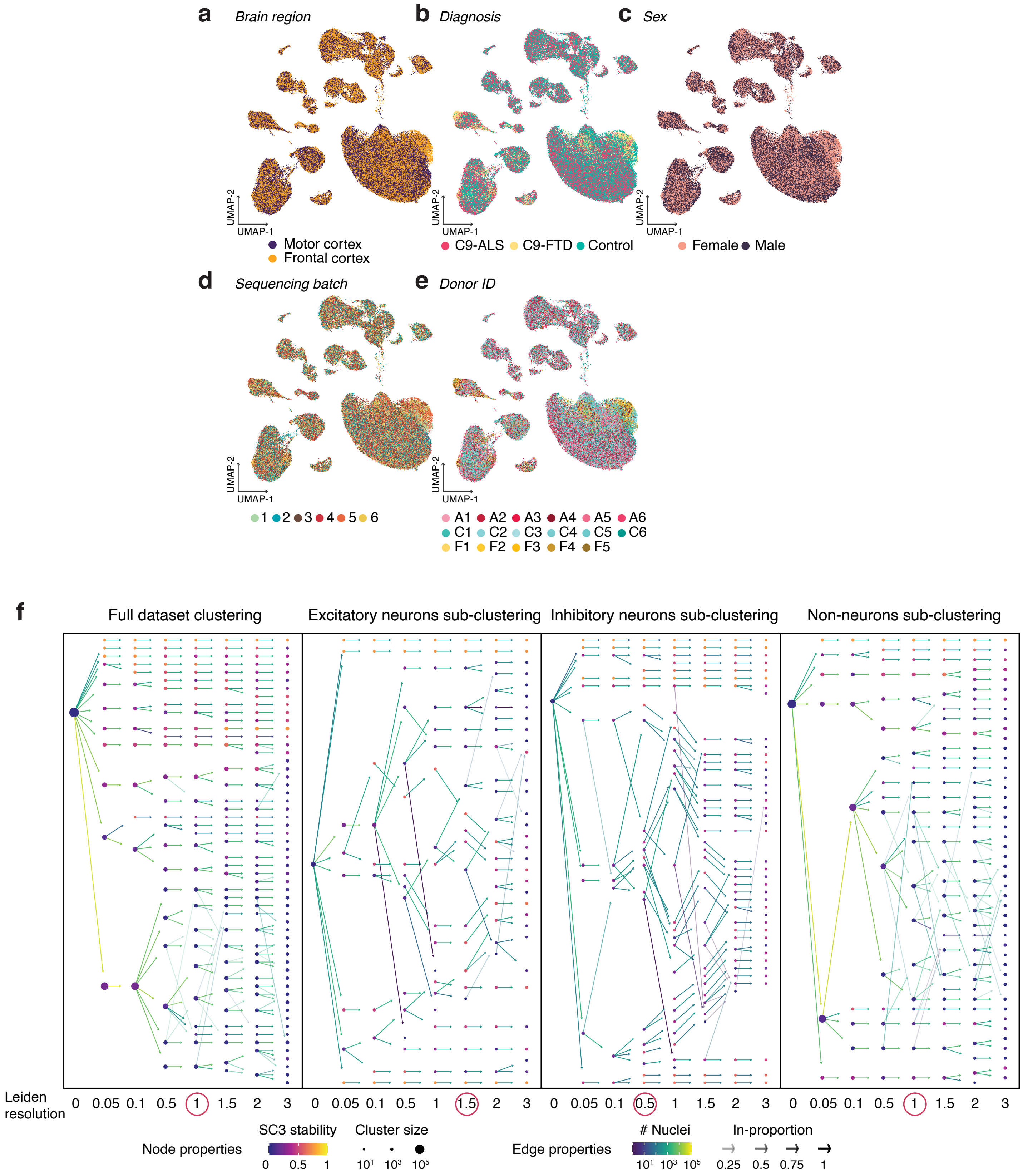

Supplementary Fig. 2. The clusters identified in snRNA-seq were not biased by known covariates and were largely stable. (a-e) Uniform manifold approximation and projection (UMAP[2]) embedding of snRNA-seq profiles (see Fig. 1a) were colored by brain region (a), diagnosis (b), donor sex (c), sequencing batch (d), or

individual donor (e). (f) Clustree[3] analysis to evaluate cluster stability with increasing Leiden resolutions from 0 to 3 in our full dataset clustering and the sub-clusterings for the three major cell classes. Clusters found at each resolution value are represented by nodes in the corresponding column. The nodes are colored by the SC3 stability index[4] and sized proportional to the number of nuclei in the cluster. The transparency of the edges is adjusted according to the in-proportion, a metric defined as the ratio between the number of nuclei on the edge and the number of nuclei in the cluster it goes toward. Low in-proportion edges tend to arise as clusters become unstable. The selected resolutions we used before cell type annotation are highlighted in red circles. Source data are provided as a Source Data file.

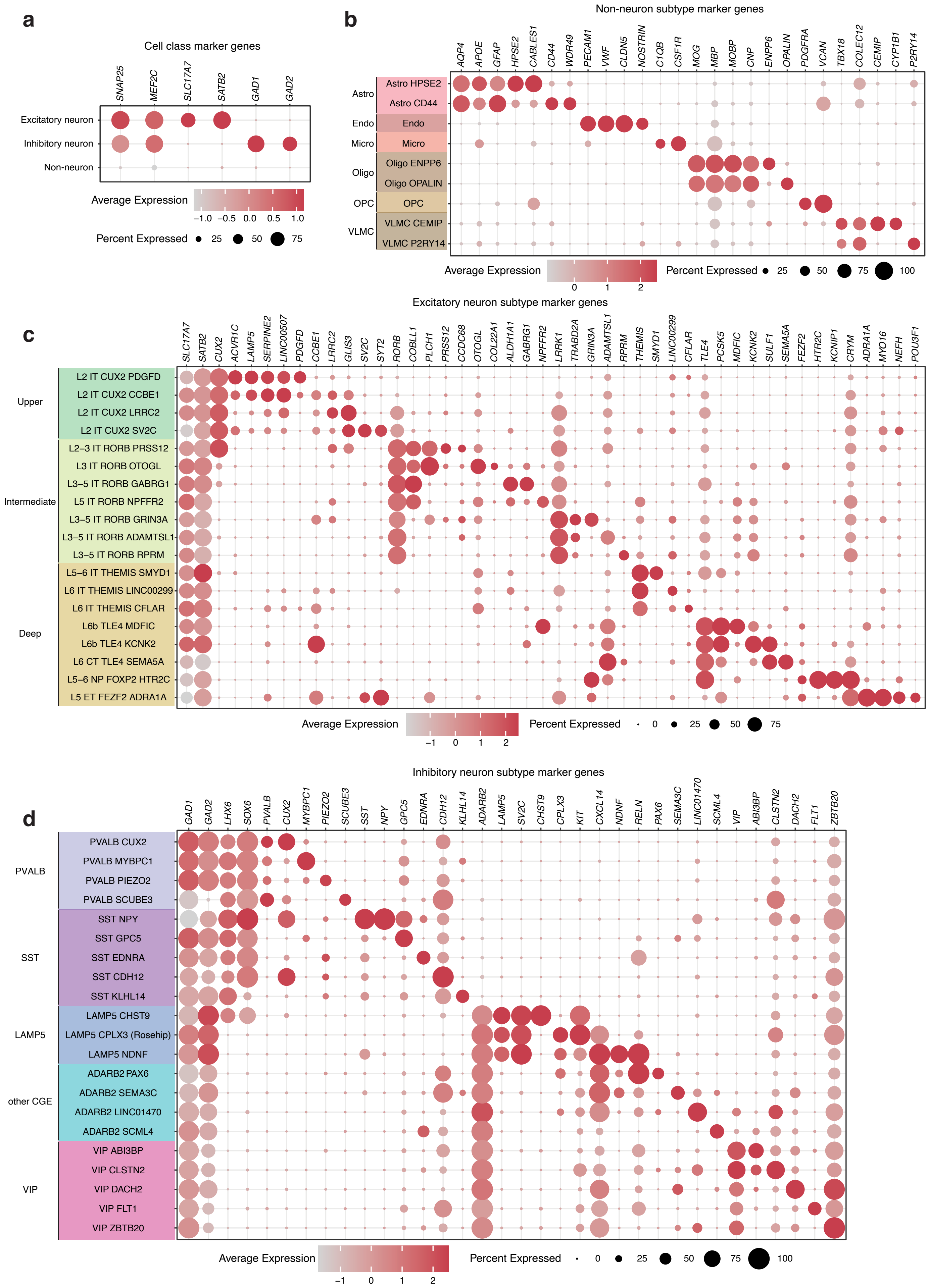
a
Cell class marker genes
SNAP25
MEF2C
SLC17A7
SATB2
GAD1
GAD2
Excitatory neuron
Inhibitory neuron
Non-neuron
Average Expression
−1.0 −0.5 0.0 0.5 1.0
Percent Expressed 25 50 75
b
Non-neuron subtype marker genes
AQP4
APOE
GFAP
HPSE2
CABLES1
CD44
WDR49
PECAM1
VWF
CLDN5
NOSTRIN
C1QB
CSF1R
MOG
MBP
MOBP
CNP
ENPP6
OPALIN
PDGFRA
VCAN
TBX18
COLEC12
CEMIP
CYP1B1
P2RY14
Astro
Astro HPSE2
Astro CD44
Endo
Micro
Oligo
Oligo ENPP6
Oligo OPALIN
OPC
VLMC
VLMC CEMIP
VLMC P2RY14
Average Expression
0 1 2
Percent Expressed 25 50 75 100
c
Excitatory neuron subtype marker genes
SLC17A7
SATB2
CUX2
ACVR1C
LAMP5
SERPINE2
LINC00507
PDGFD
CCBE1
LRRC2
GLIS3
SV2C
SYT2
RORB
COBLL1
PLCH1
PRSS12
CCDC68
OTOGL
COL22A1
ALDH1A1
GABRG1
NPFFR2
LRRK1
TRABD2A
GRIN3A
ADAMTSL1
RPRM
THEMIS
SMYD1
LINC00299
CFLAR
TLE4
PCSK5
MDFIC
KCNK2
SULF1
SEMA5A
FEZF2
HTR2C
KCNIP1
CRYM
ADRA1A
MYO16
NEFH
POU3F1
Upper
L2 IT CUX2 PDGFD
L2 IT CUX2 CCBE1
L2 IT CUX2 LRRC2
L2 IT CUX2 SV2C
Intermediate
L2−3 IT RORB PRSS12
L3 IT RORB OTOGL
L3−5 IT RORB GABRG1
L5 IT RORB NPFFR2
L3−5 IT RORB GRIN3A
L3−5 IT RORB ADAMTSL1
L3−5 IT RORB RPRM
Deep
L5−6 IT THEMIS SMYD1
L6 IT THEMIS LINC00299
L6 IT THEMIS CFLAR
L6b TLE4 MDFIC
L6b TLE4 KCNK2
L6 CT TLE4 SEMA5A
L5−6 NP FOXP2 HTR2C
L5 ET FEZF2 ADRA1A
Average Expression
−1 0 1 2
Percent Expressed 0 25 50 75
d
Inhibitory neuron subtype marker genes
GAD1
GAD2
LHX6
SOX6
PVALB
CUX2
MYBPC1
PIEZO2
SCUBE3
SST
NPY
GPC5
EDNRA
CDH12
KLHL14
ADARB2
LAMP5
SV2C
CHST9
CPLX3
KIT
CXCL14
NDNF
RELN
PAX6
SEMA3C
LINC01470
SCML4
VIP
ABI3BP
CLSTN2
DACH2
FLT1
ZBTB20
PVALB
PVALB CUX2
PVALB MYBPC1
PVALB PIEZO2
PVALB SCUBE3
SST
SST NPY
SST GPC5
SST EDNRA
SST CDH12
SST KLHL14
LAMP5
LAMP5 CHST9
LAMP5 CPLX3 (Rosehip)
LAMP5 NDNF
other CGE
ADARB2 PAX6
ADARB2 SEMA3C
ADARB2 LINC01470
ADARB2 SCML4
VIP
VIP ABI3BP
VIP CLSTN2
VIP DACH2
VIP FLT1
VIP ZBTB20
Average Expression
−1 0 1 2
Percent Expressed 0 25 50 75 100

Supplementary Fig. 3. Annotations of the identified cellular populations in snRNA-seq based on the expression of known markers. Nuclei were first categorized into excitatory and inhibitory neurons, and non-neuronal cells using cell class marker genes (a). Fine-grained cell types were then annotated using marker genes for non-neuronal cells (b), excitatory neurons (c), and inhibitory neurons (d). Major cell types (left colored bars) were defined using shared marker genes. Normalized expression is defined as the z-score of $\log$(CPM) for each gene across all cells. Dots were colored by the average normalized expression in each cell type, and dot size represents the percentage of nuclei expressing the marker genes (with non-zero counts) in the cell type. CGE, caudal ganglionic eminence; Astro, astrocytes; Endo, endothelial cells; Micro, microglia; Oligo, oligodendrocytes; OPC, oligodendrocyte precursor cells; VLMC, vascular leptomeningeal cell; IT, intratelencephalic; CT, corticothalamic; NP, near-projecting; ET, extratelencephalic. Source data are provided as a Source Data file.

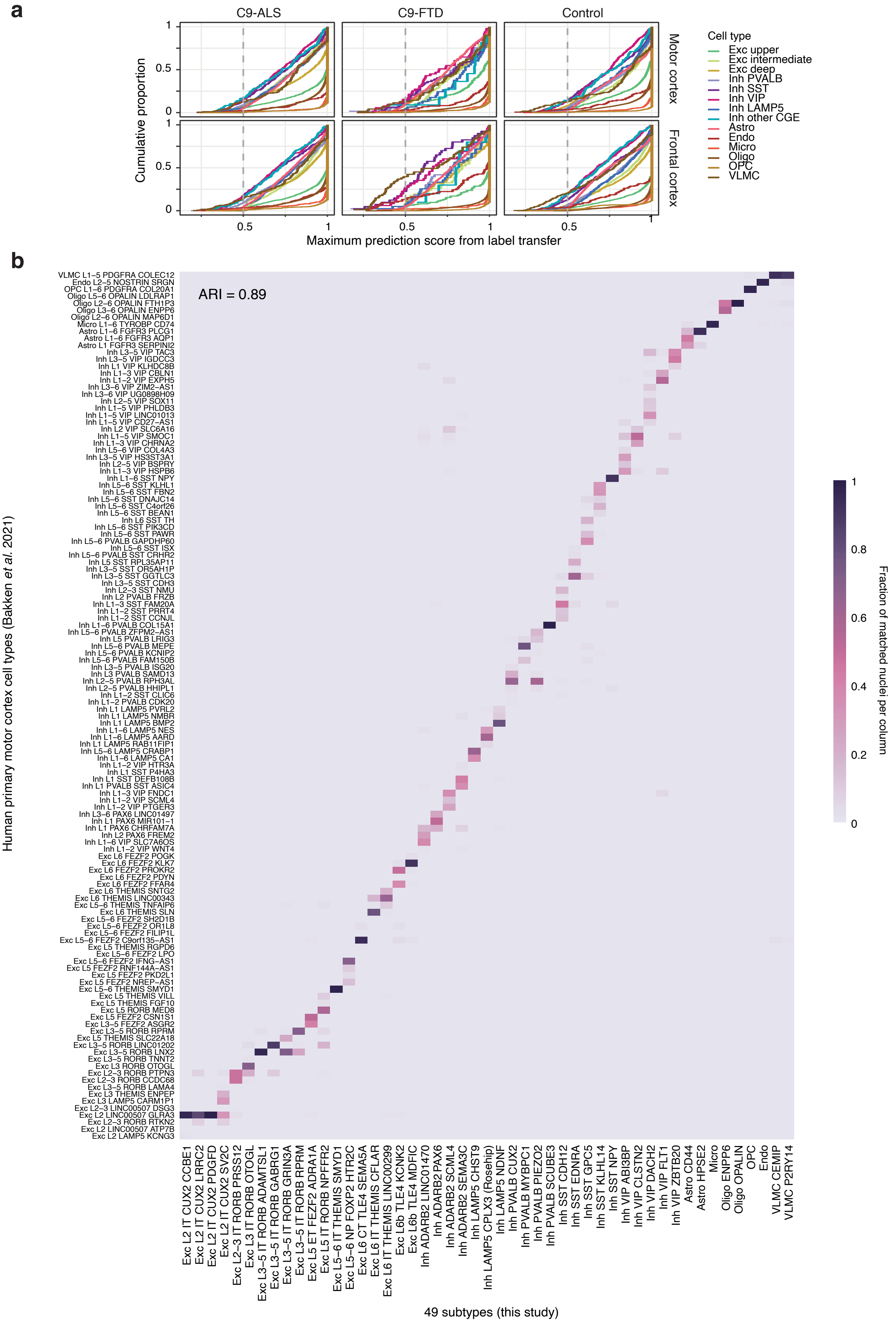
a
C9-ALS
C9-FTD
Control
Motor cortex
Frontal cortex
Cumulative proportion
0
0.5
1
Maximum prediction score from label transfer
Cell type
Exc upper
Exc intermediate
Exc deep
Inh PVALB
Inh SST
Inh VIP
Inh LAMP5
Inh other CGE
Astro
Endo
Micro
Oligo
OPC
VLMC
b
ARI = 0.89
Human primary motor cortex cell types (Bakken et al. 2021)
VLMC L1–5 PDGFRA COLEC12
Endo L2–5 NOSTRIN SRGN
OPC L1–6 PDGFRA COL20A1
Oligo L5–6 OPALIN LDLRAP1
Oligo L2–6 OPALIN FTH1P3
Oligo L3–6 OPALIN ENPP6
Oligo L2–6 OPALIN MAP6D1
Micro L1–6 TYROBP CD74
Astro L1–6 FGFR3 PLCG1
Astro L1–6 FGFR3 AQP1
Astro L1 FGFR3 SERPINI2
Inh L3–5 VIP TAC3
Inh L3–5 VIP IGDCC3
Inh L1 VIP KLHDC8B
Inh L1–3 VIP CBLN1
Inh L1–2 VIP EXPH5
Inh L3–6 VIP ZIM2–AS1
Inh L3–6 VIP UG0898H09
Inh L2–5 VIP SOX11
Inh L1–5 VIP PHLDB3
Inh L1–5 VIP LINC01013
Inh L1–5 VIP CD27–AS1
Inh L2 VIP SLC6A16
Inh L1–5 VIP SMOC1
Inh L1–3 VIP CHRNA2
Inh L5–6 VIP COL4A3
Inh L3–5 VIP HS3ST3A1
Inh L2–5 VIP BSPRY
Inh L1–3 VIP HSPB6
Inh L1–6 SST NPY
Inh L5–6 SST KLHL1
Inh L5–6 SST FBN2
Inh L5–6 SST DNAJC14
Inh L5–6 SST C4orf26
Inh L5–6 SST BEAN1
Inh L6 SST TH
Inh L5–6 SST PIK3CD
Inh L5–6 SST PAWR
Inh L5–6 PVALB GAPDHP60
Inh L5–6 SST ISX
Inh L5–6 PVALB SST CRHR2
Inh L5 SST RPL35AP11
Inh L3–5 SST OR5AH1P
Inh L3–5 SST GGTLC3
Inh L3–5 SST CDH3
Inh L2–3 SST NMU
Inh L2 PVALB FRZB
Inh L1–3 SST FAM20A
Inh L1–2 SST PRRT4
Inh L1–2 SST CCNJL
Inh L1–6 PVALB COL15A1
Inh L5–6 PVALB ZFPM2–AS1
Inh L5 PVALB LRIG3
Inh L5–6 PVALB MEPE
Inh L5–6 PVALB KCNIP2
Inh L5–6 PVALB FAM150B
Inh L3–5 PVALB ISG20
Inh L3 PVALB SAMD13
Inh L2–5 PVALB RPH3AL
Inh L2–5 PVALB HHIPL1
Inh L1–2 SST CLIC6
Inh L1–2 PVALB CDK20
Inh L1 LAMP5 PVRL2
Inh L1 LAMP5 NMBR
Inh L1 LAMP5 BMP2
Inh L1–6 LAMP5 NES
Inh L1–6 LAMP5 AARD
Inh L1 LAMP5 RAB11FIP1
Inh L5–6 LAMP5 CRABP1
Inh L1–6 LAMP5 CA1
Inh L1–2 VIP HTR3A
Inh L1 SST P4HA3
Inh L1 SST DEFB108B
Inh L1 PVALB SST ASIC4
Inh L1–3 VIP FNDC1
Inh L1–2 VIP SCML4
Inh L1–2 VIP PTGER3
Inh L3–6 PAX6 LINC01497
Inh L1 PAX6 MIR101–1
Inh L1 PAX6 CHRFAM7A
Inh L2 PAX6 FREM2
Inh L1–6 VIP SLC7A6OS
Inh L1–2 VIP WNT4
Exc L6 FEZF2 POGK
Exc L6 FEZF2 KLK7
Exc L6 FEZF2 PROKR2
Exc L6 FEZF2 PDYN
Exc L6 FEZF2 FFAR4
Exc L6 THEMIS SNTG2
Exc L6 THEMIS LINC00343
Exc L5–6 THEMIS TNFAIP6
Exc L6 THEMIS SLN
Exc L5–6 FEZF2 SH2D1B
Exc L5–6 FEZF2 OR1L8
Exc L5–6 FEZF2 FILIP1L
Exc L5–6 FEZF2 C9orf135–AS1
Exc L5 THEMIS RGPD6
Exc L5–6 FEZF2 LPO
Exc L5–6 FEZF2 IFNG–AS1
Exc L5 FEZF2 RNF144A–AS1
Exc L5 FEZF2 PKD2L1
Exc L5 FEZF2 NREP–AS1
Exc L5–6 THEMIS SMYD1
Exc L5 THEMIS VILL
Exc L5 THEMIS FGF10
Exc L5 RORB MED8
Exc L5 FEZF2 CSN1S1
Exc L3–5 FEZF2 ASGR2
Exc L3–5 RORB RPRM
Exc L5 THEMIS SLC22A18
Exc L3–5 RORB LINC01202
Exc L3–5 RORB LNX2
Exc L3–5 RORB TNNT2
Exc L3 RORB OTOGL
Exc L2–3 RORB PTPN3
Exc L2–3 RORB CCDC68
Exc L3–5 RORB LAMA4
Exc L3 THEMIS ENPEP
Exc L3 LAMP5 CARM1P1
Exc L2–3 LINC00507 DSG3
Exc L2 LINC00507 GLRA3
Exc L2–3 RORB RTKN2
Exc L2 LINC00507 ATP7B
Exc L2 LAMP5 KCNG3
Exc L2 IT CUX2 CCBE1
Exc L2 IT CUX2 LRRC2
Exc L2 IT CUX2 PDGFD
Exc L2 IT CUX2 SV2C
Exc L2–3 IT RORB PRSS12
Exc L3 IT RORB OTOGL
Exc L3–5 IT RORB ADAMTSL1
Exc L3–5 IT RORB GABRG1
Exc L3–5 IT RORB GRIN3A
Exc L3–5 IT RORB RPRM
Exc L5 ET FEZF2 ADRA1A
Exc L5 IT RORB NPFFR2
Exc L5–6 IT THEMIS SMYD1
Exc L5–6 NP FOXP2 HTR2C
Exc L6 CT TLE4 SEMA5A
Exc L6 IT THEMIS CFLAR
Exc L6 IT THEMIS LINC00299
Exc L6b TLE4 KCNK2
Exc L6b TLE4 MDFIC
Inh ADARB2 LINC01470
Inh ADARB2 PAX6
Inh ADARB2 SCML4
Inh ADARB2 SEMA3C
Inh LAMP5 CHST9
Inh LAMP5 CPLX3 (Rosehip)
Inh LAMP5 NDNF
Inh PVALB CUX2
Inh PVALB MYBPC1
Inh PVALB PIEZO2
Inh PVALB SCUBE3
Inh SST CDH12
Inh SST EDNRA
Inh SST GPC5
Inh SST KLHL14
Inh SST NPY
Inh VIP ABI3BP
Inh VIP CLSTN2
Inh VIP DACH2
Inh VIP FLT1
Inh VIP ZBTB20
Astro CD44
Astro HPSE2
Micro
Oligo ENPP6
Oligo OPALIN
OPC
Endo
VLMC CEMIP
VLMC P2RY14
49 subtypes (this study)
Fraction of matched nuclei per column
0
0.2
0.4
0.6
0.8
1

Supplementary Fig. 4. Comparison of the cell types identified in our snRNA-seq dataset with a previously published dataset. (a) Cumulative proportion of the maximum prediction score across nuclei from the label transfer analysis using the published human primary motor cortex cell types[5] as the reference dataset (see Methods). Curves are grouped and colored by diagnosis, brain region, and the major cell types identified in our study. (b) The annotations in Fig. 1 and Supplementary Fig. 3 were consistent with cell types in the study of the human motor cortex[5]. ARI, adjusted Rand index. Source data are provided as a Source Data file.

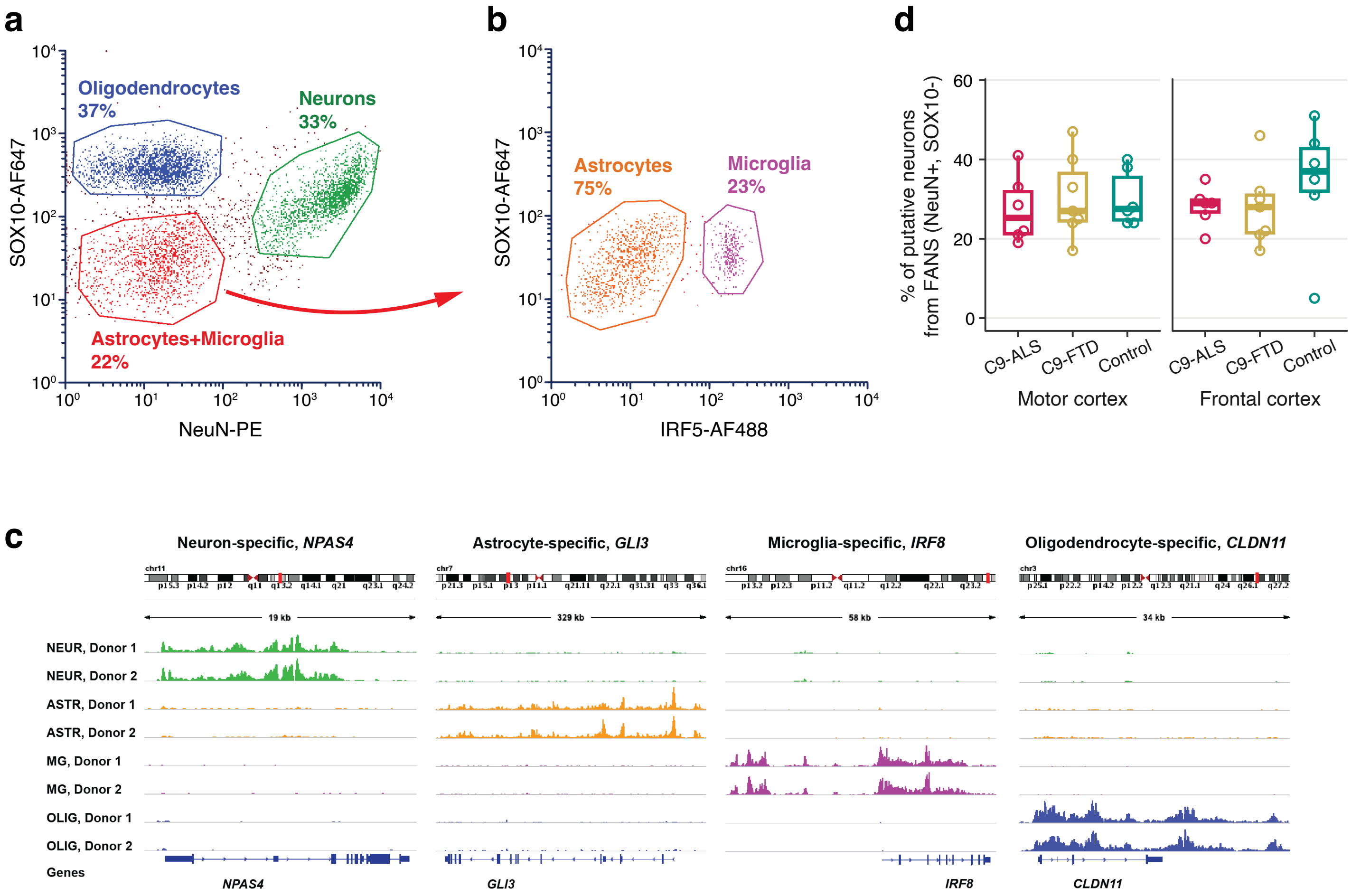

Supplementary Fig. 5. Fluorescence-activated nuclei sorting (FANS) isolation of nuclei of major brain cell types. (a,b) Sequential FANS gating procedure to isolate nuclei from cell populations. (a) Three cell populations were isolated for bulk RNA-seq (Figs. 2e-f and 6f): neurons (NeuN+), oligodendrocyte lineage cells (NeuN–/Sox10+, consisting mainly of mature oligodendrocytes and a smaller population of OPCs), and other glia (NeuN–/Sox10–, mostly consisting of microglia and astrocytes). Anti-NeuN and anti-SOX10 antibodies were used to separate these three populations. (b) For H3K27ac ChIP-seq (Fig. 5), the NeuN–/Sox10– population was further split to isolate microglia (IRF5+), and astrocytes (IRF5–). (c) Validation of FANS-separated neurons, oligodendrocyte lineage cells, microglia and astrocytes using H3K27ac ChIP-seq. Signals from two donors are shown. In each population, H3K27ac signal enrichment was detected for known cell-type-specific genes. NEUR, neurons; ASTR, astrocytes; OLIG, oligodendrocyte lineage; MG, microglia. (d) Percentage of putative neurons (NeuN+SOX10- nuclei) from FANS. Circles represent biologically independent individual donors; N = 6 C9-ALS, 6 control and 7 C9-FTD samples, respectively. In each box plot, the lower and upper hinges correspond to the first and third quartiles; the whiskers extend 1.5 * IQR (interquartile range) away from the hinges; and the center denotes the median. No significant differences were found in any pairwise comparison between diagnosis groups (two-sided Welch's t-test, p=0.41-0.97). Source data are provided as a Source Data file.

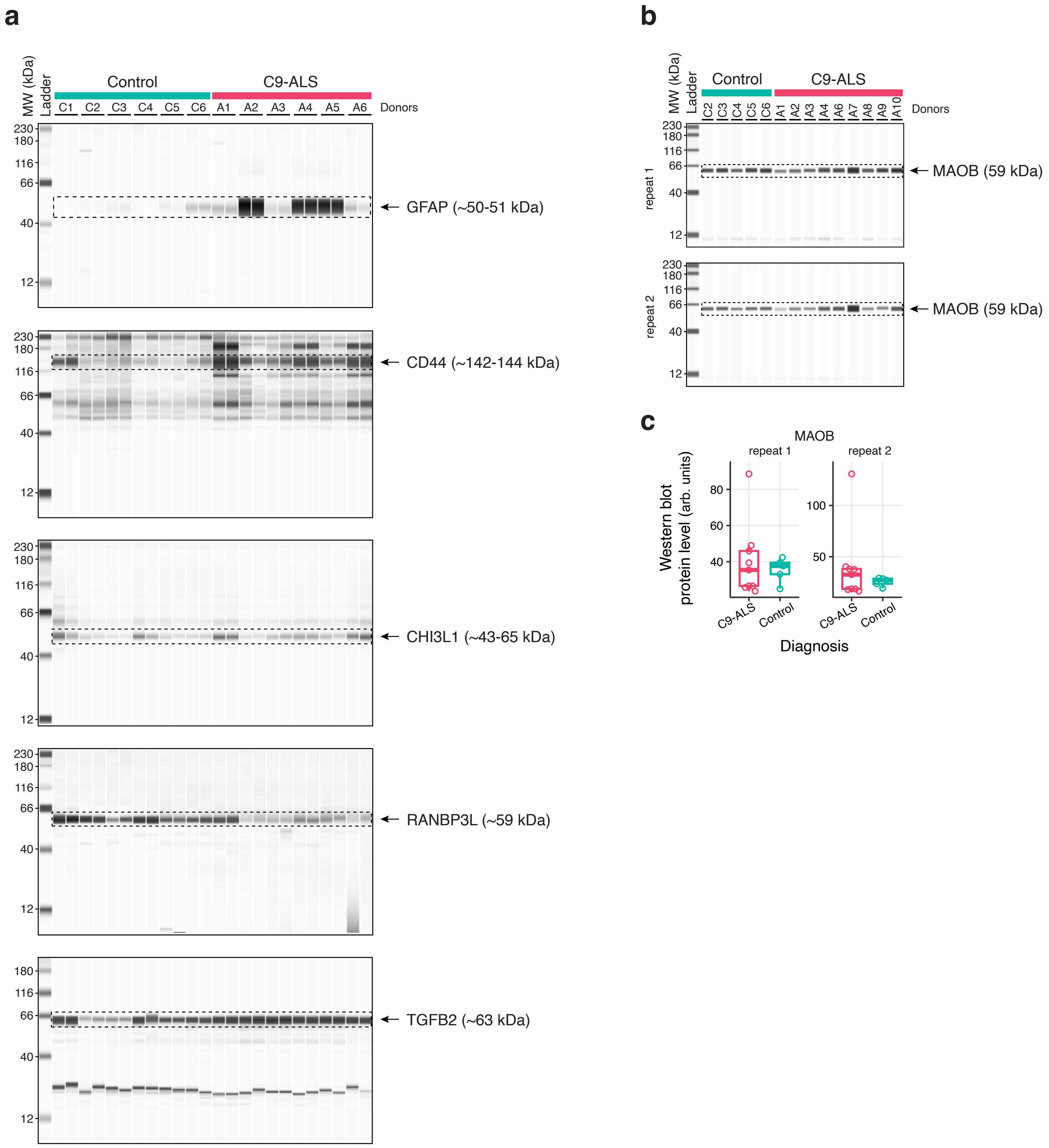

Supplementary Fig. 6. Quantification of proteins encoded by genes dysregulated in C9-ALS astrocytes using automated Western blot analysis. (a) Protein levels were determined in selected DE genes identified in astrocytes in our snRNA-seq analysis. Bulk motor cortex tissues (~80-100mg) from six C9-ALS and six control donors were used. Automated capillary Western blot analysis was performed using the ProteinSimple Jess-Wes System (Methods). For each protein, an experiment was run once with two technical replicates for

each sample. See Fig. 3c for the quantifications of protein levels. A two-sided Welch's t-test was used to compare the immunoreactive signals between C9-ALS and control, considering the averaged signals between two replicates for each donor as an observation. GFAP, CD44, and TGFB2 proteins were upregulated ($p = 0.033, 0.012, 0.033$ respectively) in C9-ALS motor cortex, whereas RANBP3L protein trended in the expected direction ($p = 0.063$) and CHI3L1 was not significant ($p = 0.303$). (b) MAOB protein levels from nine C9-ALS and five control donors. Each sample was assessed in two repeated experiments. (c) Quantifications of protein levels in (b). Arb. units, arbitrary units. Circles represent the signal for each biologically independent individual donor; N = 9 C9-ALS and 5 control samples. In each box plot, the lower and upper hinges correspond to the first and third quartiles; the whiskers extend 1.5 * IQR (interquartile range) away from the hinges; and the center denotes the median. For each repeat experiment, a two-sided Welch's t-test was used to compare the immunoreactive signals between C9-ALS and control. No significant changes of MAOB protein levels were detected ($p=0.549$ and 0.297). See Supplementary Dataset 10 for raw values. Source data are provided as a Source Data file.

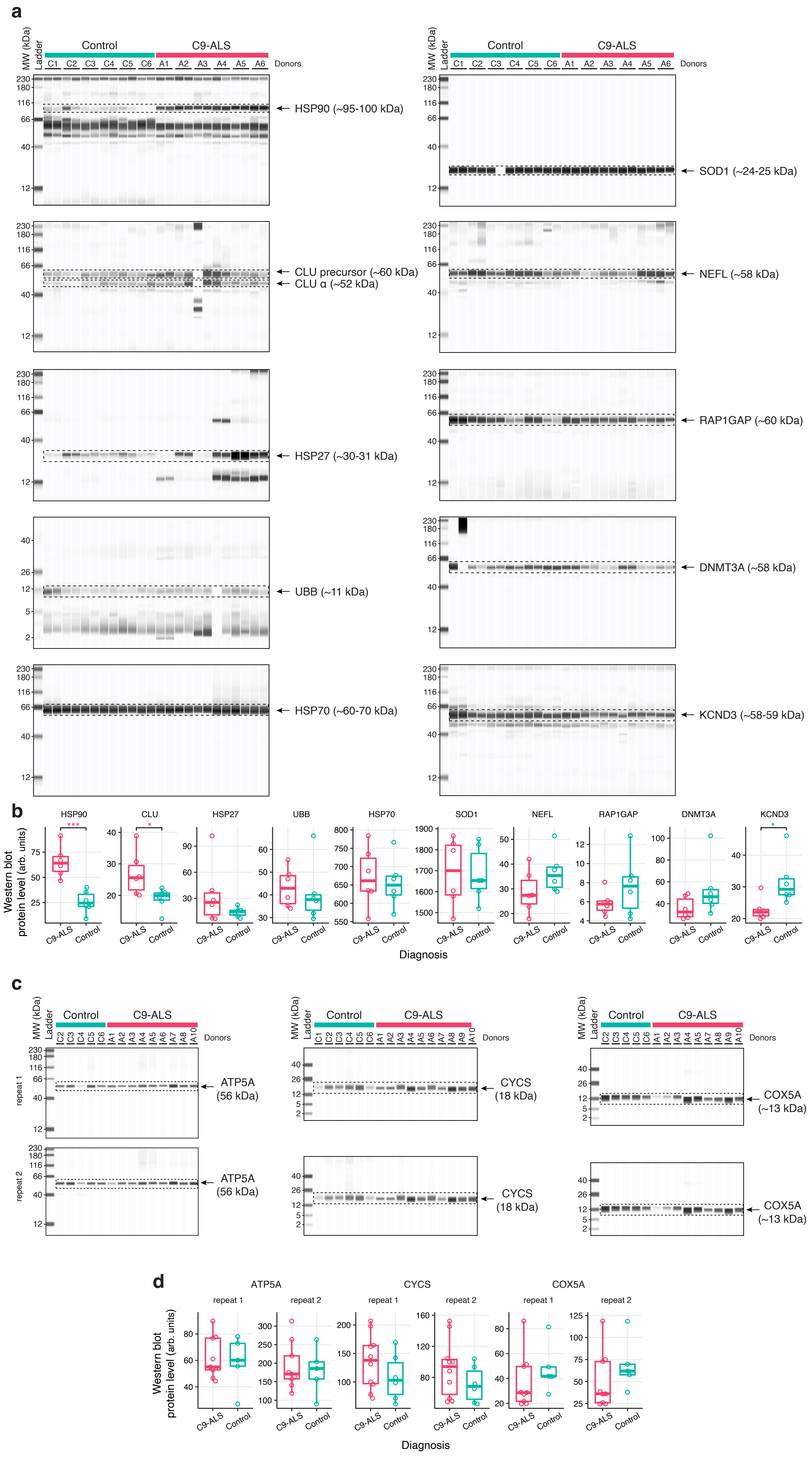
a
Control
C9-ALS
MW (kDa)
Ladder
C1 C2 C3 C4 C5 C6 A1 A2 A3 A4 A5 A6
Donors
HSP90 (~95-100 kDa)
SOD1 (~24-25 kDa)
CLU precursor (~60 kDa)
CLU α (~52 kDa)
NEFL (~58 kDa)
HSP27 (~30-31 kDa)
RAP1GAP (~60 kDa)
UBB (~11 kDa)
DNMT3A (~58 kDa)
HSP70 (~60-70 kDa)
KCND3 (~58-59 kDa)
b
HSP90
CLU
HSP27
UBB
HSP70
SOD1
NEFL
RAP1GAP
DNMT3A
KCND3
***
*
*
Western blot protein level (arb. units)
C9-ALS
Control
Diagnosis
c
Control
C9-ALS
C2 C3 C4 C5 C6 A1 A2 A3 A4 A5 A6 A7 A8 A10
C1 C2 C3 C4 C5 C6 A1 A2 A3 A4 A5 A6 A7 A8 A9 A10
C2 C3 C4 C5 C6 A1 A2 A3 A4 A5 A7 A8 A9 A10
Donors
repeat 1
repeat 2
ATP5A (56 kDa)
CYCS (18 kDa)
COX5A (~13 kDa)
d
ATP5A
CYCS
COX5A
repeat 1
repeat 2
Western blot protein level (arb. units)
C9-ALS
Control
Diagnosis

Supplementary Fig. 7. Quantification of proteins encoded by genes dysregulated in C9-ALS excitatory neurons using automated Western blot analysis. (a) Same as Supplementary Fig. 6a but for selected DE genes identified in excitatory neurons with our snRNA-seq dataset. (b) Quantification of protein levels in (a) with automated Western blot analysis. Arb. units, arbitrary units. Circles represent the average signal across duplicates for each donor; N = 6 C9-ALS and 6 control biologically independent samples. *, two-sided Welch's t-test $p < 0.05$; ***, $p < 0.001$. HSP90 and CLU proteins were upregulated ($p = 6.35e-4$ and 0.041, respectively), KCND3 was downregulated ($p = 0.036$), and HSP27, RAP1GAP and DNMT3A proteins were not significant ($p = 0.226$, 0.238 and 0.163, respectively). None of the tested genes had a change in protein abundance in the opposite direction from the mRNA expression change. (c) ATP5A, CYCS and COX5A protein levels (n=10 C9-ALS and 6 control donors for CYCS; n=9 C9-ALS and 5 control donors for ATP5A and COX5A). Each sample was assessed in two repeated experiments. (d) Quantifications of protein levels in (c). Arb. units, arbitrary units. Circles represent the signal for each donor; N = 10 C9-ALS and 6 control donors for CYCS, and N = 9 C9-ALS and 5 control donors for ATP5A and COX5A. For each repeat experiment, a two-sided Welch's t-test was used to compare the immunoreactive signals between C9-ALS and control. No significant changes of ATP5A, CYCS or COX5A protein levels were detected (p=0.206-0.757). In each box plot in panels (b) and (d), the lower and upper hinges correspond to the first and third quartiles; the whiskers extend 1.5 * IQR (interquartile range) away from the hinges; and the center denotes the median. See Supplementary Dataset 10 for raw values. Source data are provided as a Source Data file.

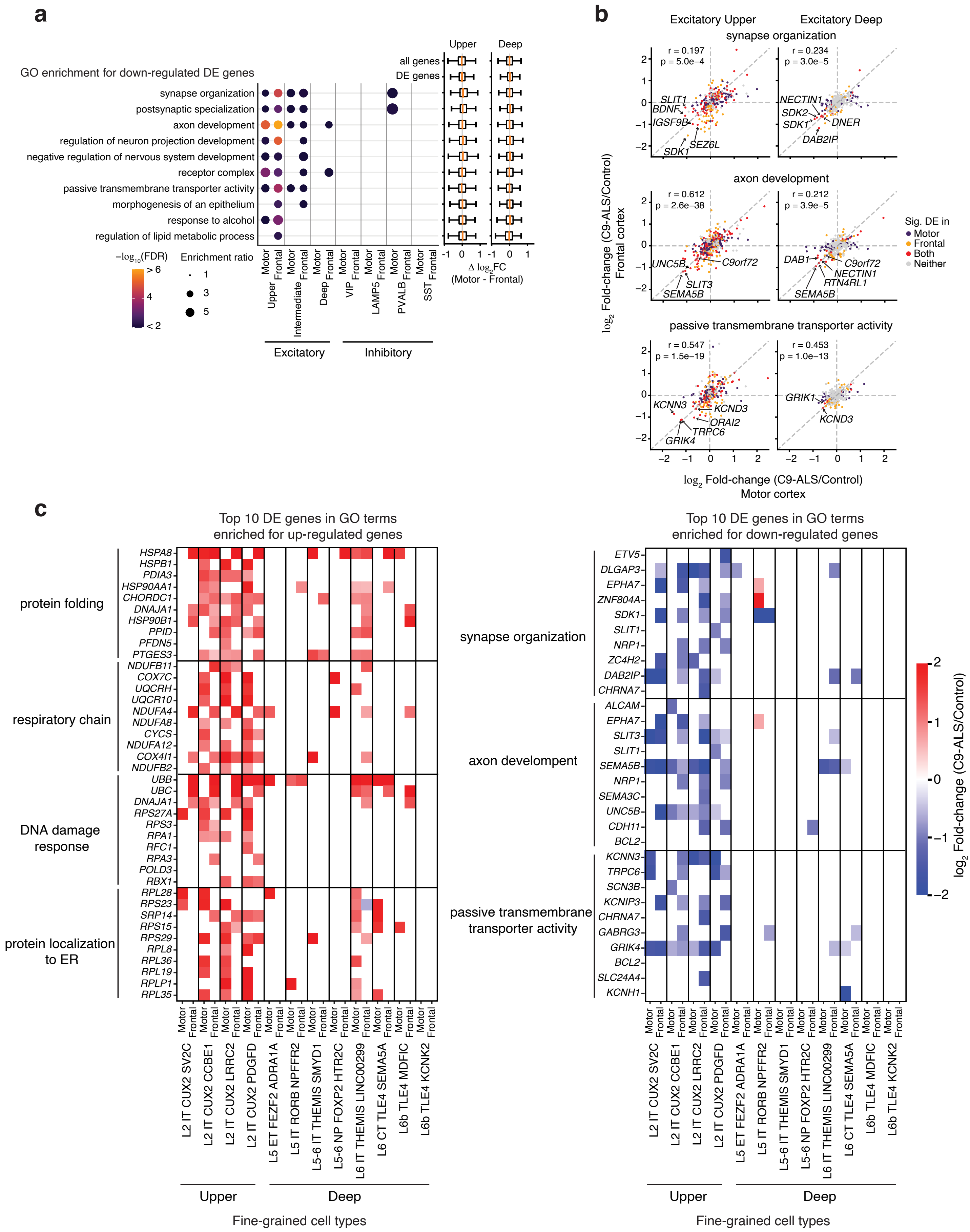
a
GO enrichment for down-regulated DE genes
synapse organization
postsynaptic specialization
axon development
regulation of neuron projection development
negative regulation of nervous system development
receptor complex
passive transmembrane transporter activity
morphogenesis of an epithelium
response to alcohol
regulation of lipid metabolic process
all genes
DE genes
Upper
Deep
−log10(FDR)
> 6
4
< 2
Enrichment ratio
1
3
5
Motor
Frontal
Upper
Intermediate
Deep
VIP
LAMP5
PVALB
SST
Excitatory
Inhibitory
Δ log2FC
(Motor - Frontal)
b
Excitatory Upper
Excitatory Deep
synapse organization
r = 0.197
p = 5.0e−4
r = 0.234
p = 3.0e−5
SLIT1
BDNF
IGSF9B
SEZ6L
SDK1
NECTIN1
SDK2
SDK1
DNER
DAB2IP
axon development
r = 0.612
p = 2.6e−38
r = 0.212
p = 3.9e−5
UNC5B
C9orf72
SLIT3
SEMA5B
DAB1
C9orf72
NECTIN1
RTN4RL1
SEMA5B
Sig. DE in
Motor
Frontal
Both
Neither
passive transmembrane transporter activity
r = 0.547
p = 1.5e−19
r = 0.453
p = 1.0e−13
KCNN3
KCND3
ORAI2
TRPC6
GRIK4
GRIK1
KCND3
log2 Fold-change (C9-ALS/Control)
Frontal cortex
log2 Fold-change (C9-ALS/Control)
Motor cortex
c
Top 10 DE genes in GO terms
enriched for up-regulated genes
protein folding
HSPA8
HSPB1
PDIA3
HSP90AA1
CHORDC1
DNAJA1
HSP90B1
PPID
PFDN5
PTGES3
respiratory chain
NDUFB11
COX7C
UQCRH
UQCR10
NDUFA4
NDUFA8
CYCS
NDUFA12
COX4I1
NDUFB2
DNA damage
response
UBB
UBC
DNAJA1
RPS27A
RPS3
RPA1
RFC1
RPA3
POLD3
RBX1
protein localization
to ER
RPL28
RPS23
SRP14
RPS15
RPS29
RPL8
RPL36
RPL19
RPLP1
RPL35
Top 10 DE genes in GO terms
enriched for down-regulated genes
synapse organization
ETV5
DLGAP3
EPHA7
ZNF804A
SDK1
SLIT1
NRP1
ZC4H2
DAB2IP
CHRNA7
axon development
ALCAM
EPHA7
SLIT3
SLIT1
SEMA5B
NRP1
SEMA3C
UNC5B
CDH11
BCL2
passive transmembrane
transporter activity
KCNN3
TRPC6
SCN3B
KCNIP3
CHRNA7
GABRG3
GRIK4
BCL2
SLC24A4
KCNH1
log2 Fold-change (C9-ALS/Control)
L2 IT CUX2 SV2C
L2 IT CUX2 CCBE1
L2 IT CUX2 LRRC2
L2 IT CUX2 PDGFD
L5 ET FEZF2 ADRA1A
L5 IT RORB NPFFR2
L5-6 IT THEMIS SMYD1
L5-6 NP FOXP2 HTR2C
L6 IT THEMIS LINC00299
L6 CT TLE4 SEMA5A
L6b TLE4 MDFIC
L6b TLE4 KCNK2
Upper
Deep
Fine-grained cell types

Supplementary Fig. 8. Functional enrichment analysis of differentially expressed (DE) genes in upper- and deep-layer excitatory neurons. (a) Left panel: Top gene ontology (GO) terms enriched for genes downregulated in upper and/or deep layer excitatory neurons. Enrichments of the same terms for downregulated DE genes in other neuronal cell types are shown for comparison. Enriched GO categories (FDR<0.01) were selected by affinity propagation. Right panel: Differences in the C9-ALS vs. control fold-changes between motor and frontal cortex ($\Delta \log_2$FC = $\log_2$FC in motor cortex - $\log_2$FC in frontal cortex). The boxes denote the distribution of these differences ($\Delta \log_2$FC) for all expressed genes in each GO category. As background comparisons, the distributions of these differences for all expressed genes (labeled as "all genes") and for all ALS vs. control DE genes (labeled as "DE gene") are shown on top. Two-sided Welch's t-tests were used to test whether the $\Delta \log_2$FC in each group of genes were significantly different from the $\Delta \log_2$FC of the "all genes" control set. No significant differences were found. In each box plot, the lower and upper hinges correspond to the first and third quartiles; the whiskers extend 1.5 * IQR (interquartile range) away from the hinges; and the center denotes the median. Exact N numbers and p-values for this analysis are provided in Supplementary Dataset 5. (b) Comparison of effects in motor vs. frontal cortices for GO categories exemplifying processes/structures that are specific for neurons (synapse organization, axon development), or important for neuronal function (passive transmembrane transport activity). r, Pearson correlation coefficient. (c) mRNA expression fold-change (C9-ALS vs. control) of top 10 DE genes for GO terms enriched for upregulated DE genes (left panel) and downregulated genes (right panel) in fine-grained upper- and deep-layer excitatory neurons. See Supplementary Dataset 5 for the full list of GO enrichment results. Source data are provided as a Source Data file.

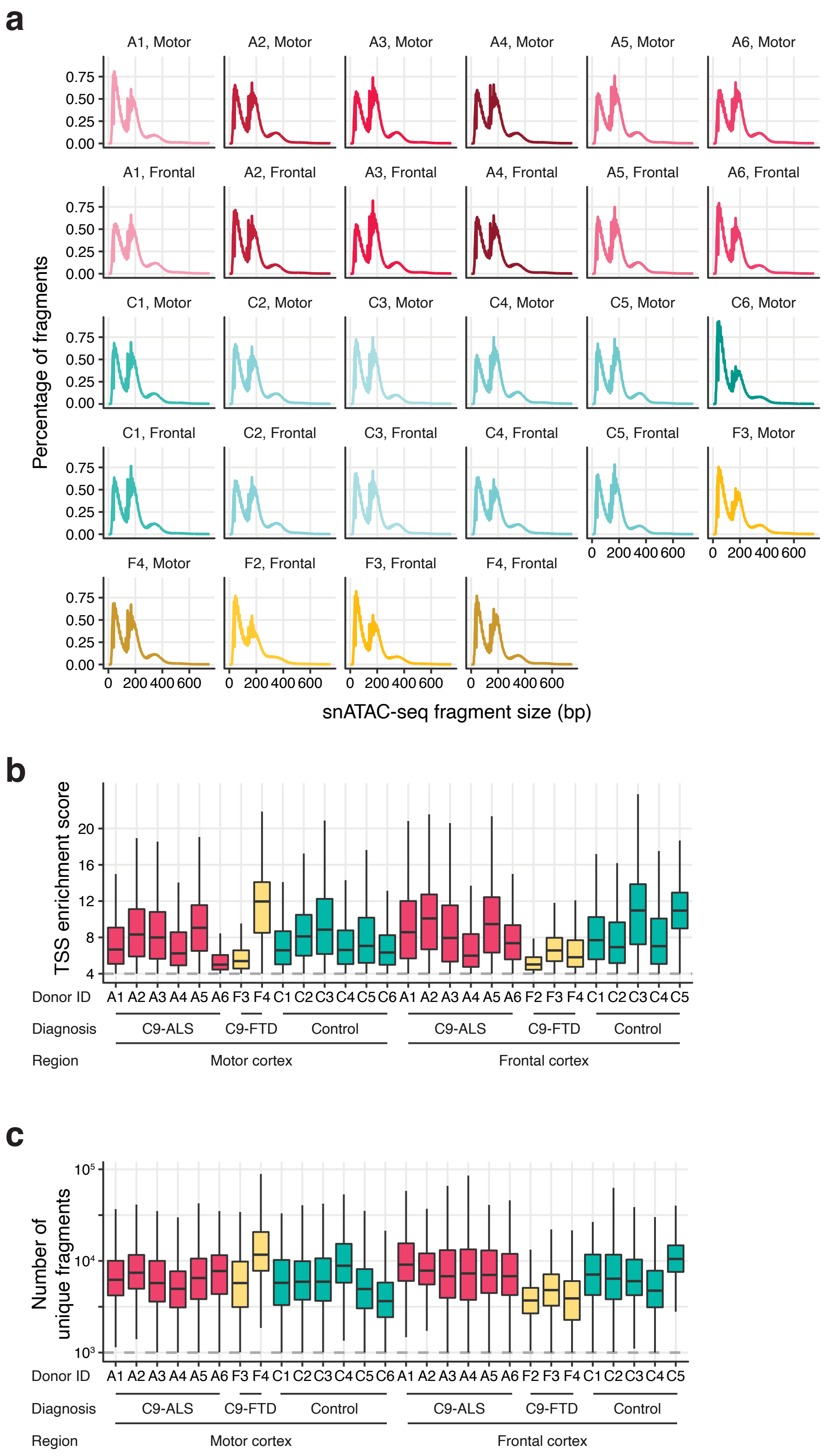

Supplementary Fig. 9. QC metrics used in the snATAC-seq data pre-processing. (a) Distribution of snATAC-seq fragment sizes in each sample that passed QC, showing periodicity related to nucleosome spacing. (b-c) Box-and-whisker plots to show the distribution of transcription start site (TSS) enrichment score (b) and number of unique fragments (c) across nuclei grouped by samples. In each box plot, the lower and upper hinges correspond to the first and third quartiles; the whiskers extend 1.5 * IQR (interquartile range) away from the hinges; and the center denotes the median. N numbers for each box plot in panels (b) and (c)

can be derived from the full metadata information of each nucleus from the snATAC-seq data provided in Supplementary Dataset 6. Source data are provided as a Source Data file.

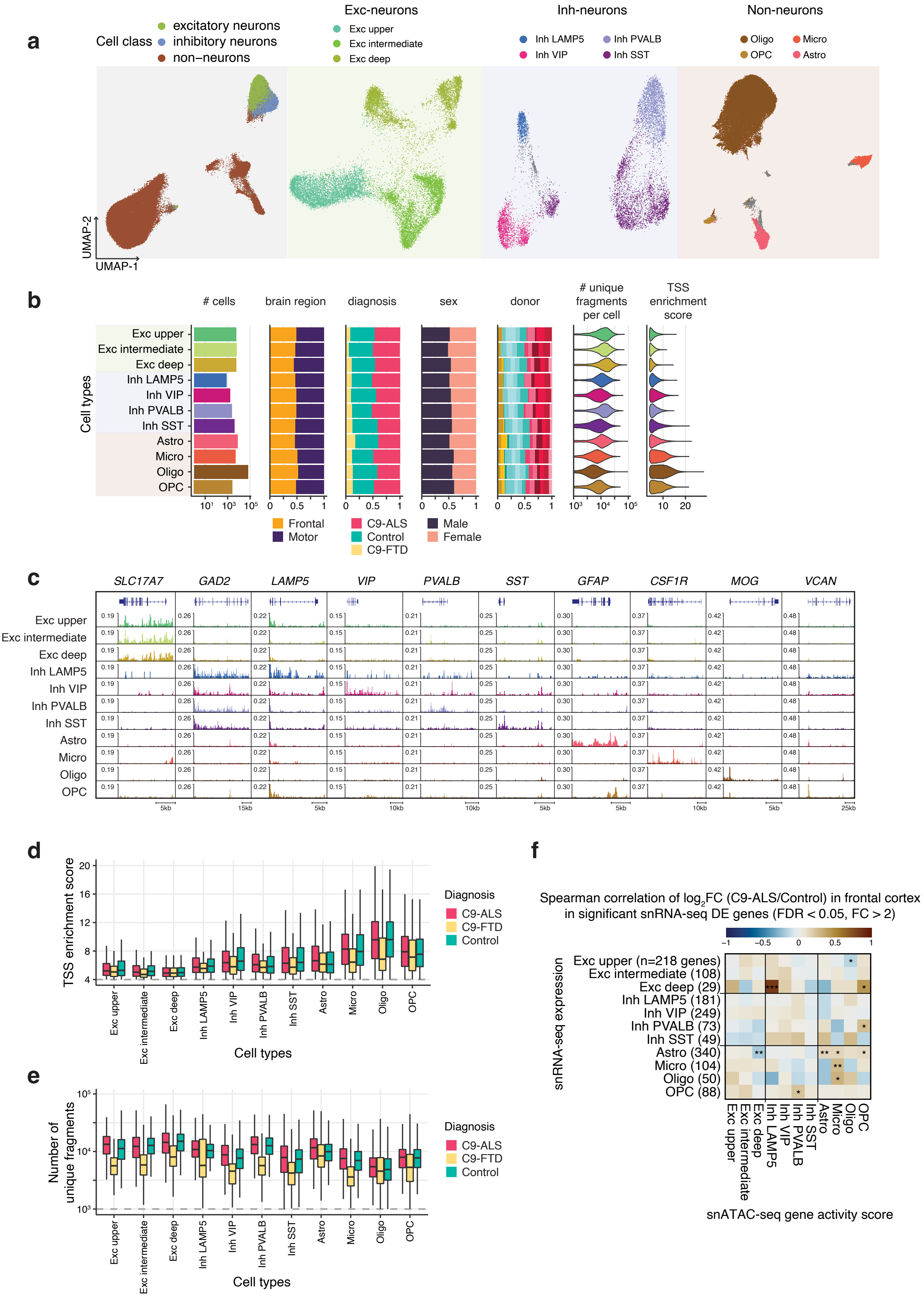
a
Cell class
excitatory neurons
inhibitory neurons
non–neurons
Exc-neurons
Exc upper
Exc intermediate
Exc deep
Inh-neurons
Inh LAMP5
Inh VIP
Inh PVALB
Inh SST
Non-neurons
Oligo
OPC
Micro
Astro
UMAP-2
UMAP-1
b
cells
brain region
diagnosis
sex
donor
unique fragments per cell
TSS enrichment score
Cell types
Exc upper
Exc intermediate
Exc deep
Inh LAMP5
Inh VIP
Inh PVALB
Inh SST
Astro
Micro
Oligo
OPC
Frontal
Motor
C9-ALS
Control
C9-FTD
Male
Female
c
SLC17A7
GAD2
LAMP5
VIP
PVALB
SST
GFAP
CSF1R
MOG
VCAN
5kb
15kb
5kb
10kb
10kb
5kb
5kb
10kb
5kb
25kb
d
TSS enrichment score
Cell types
Diagnosis
C9-ALS
C9-FTD
Control
e
Number of unique fragments
Cell types
Diagnosis
C9-ALS
C9-FTD
Control
f
Spearman correlation of log2FC (C9-ALS/Control) in frontal cortex in significant snRNA-seq DE genes (FDR < 0.05, FC > 2)
snRNA-seq expression
Exc upper (n=218 genes)
Exc intermediate (108)
Exc deep (29)
Inh LAMP5 (181)
Inh VIP (249)
Inh PVALB (73)
Inh SST (49)
Astro (340)
Micro (104)
Oligo (50)
OPC (88)
snATAC-seq gene activity score

Supplementary Fig. 10. The epigenetic landscape of C9-ALS brain cells determined by snATAC-seq. (a) Clustering n=109,198 high-quality snATAC-seq profiles (TSS enrichment ≥ 4, unique fragments ≥ 1,000 per cell) identified 11 major brain cell types. The clusters were annotated by transferring labels from the snRNA-seq data (see Methods). (b) Major cell types from snATAC-seq were distributed across brain regions, diagnosis groups, sex, and donors. TSS, transcription start site. (c) snATAC-seq signal at cell-type-specific marker genes. Track height represents pseudo-bulk counts normalized by reads in TSS. (d-e) Box-and-whisker plots to show the distribution of TSS enrichment score (d) and number of unique fragments (e) across nuclei grouped by major cell types. In each box plot, the lower and upper hinges correspond to the first and third quartiles; the whiskers extend 1.5 * IQR (interquartile range) away from the hinges; and the center denotes the median. N numbers for each box plot in panels (d) and (e) can be derived from the full metadata information of each nucleus from the snATAC-seq data provided in Supplementary Dataset 6. (f) Spearman correlation of the C9-ALS vs. control fold-change (FC) for snRNA expression vs. snATAC gene activity score in frontal cortex; the corresponding analysis for motor cortex is shown in Fig. 5e. The analysis was performed for strongly DE genes (FC > 2) in each major cell type in frontal cortex. Two-sided Spearman's rank correlation test: *, $p < 0.05$; **, $p < 0.01$; ***, $p < 0.001$. Exact values of r and p are provided in Supplementary Dataset 12. Source data are provided as a Source Data file.

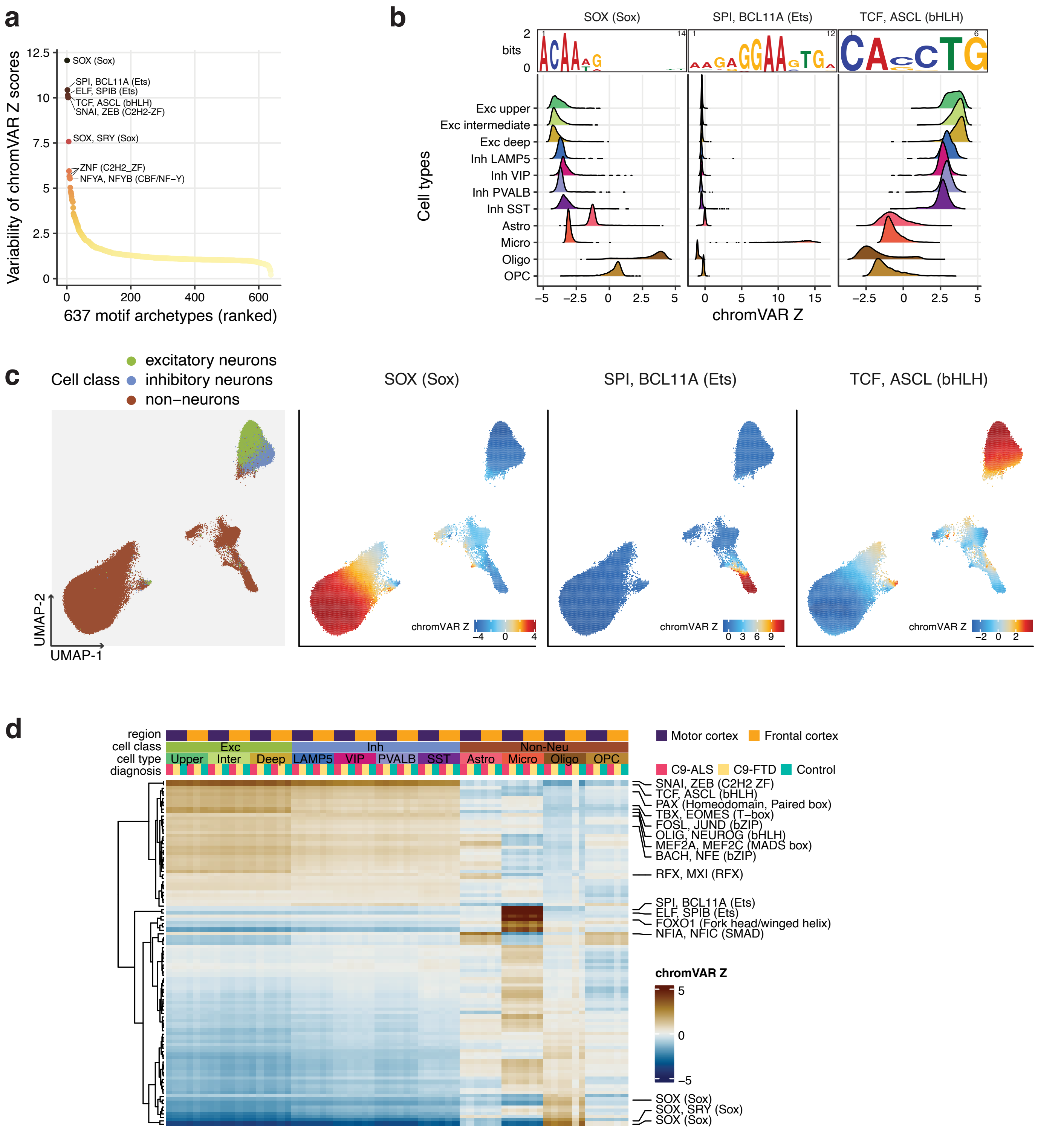

Supplementary Fig. 11. ChromVAR scores in non-redundant transcription factor (TF) motif archetypes. (a) Variability (standard deviation) of the ChromVAR[6] Z scores across all nuclei for TF motif archetypes (see Methods). (b) Distribution of ChromVAR Z score across nuclei from each major cell type for three representative TF motif archetypes with high variability. Sequence logos of the motif archetypes are shown on top. (c) ChromVAR Z scores of motif archetypes for each cell are displayed on the UMAP embedding. (d) Heatmap of average ChromVAR Z scores across groups of nuclei (columns) by major cell types, brain region and diagnosis, for the top 100 motif archetypes (rows) with highest variability across all nuclei. Top 3 cell-type-specific motif archetypes for each cell type are labeled. Source data are provided as a Source Data file.

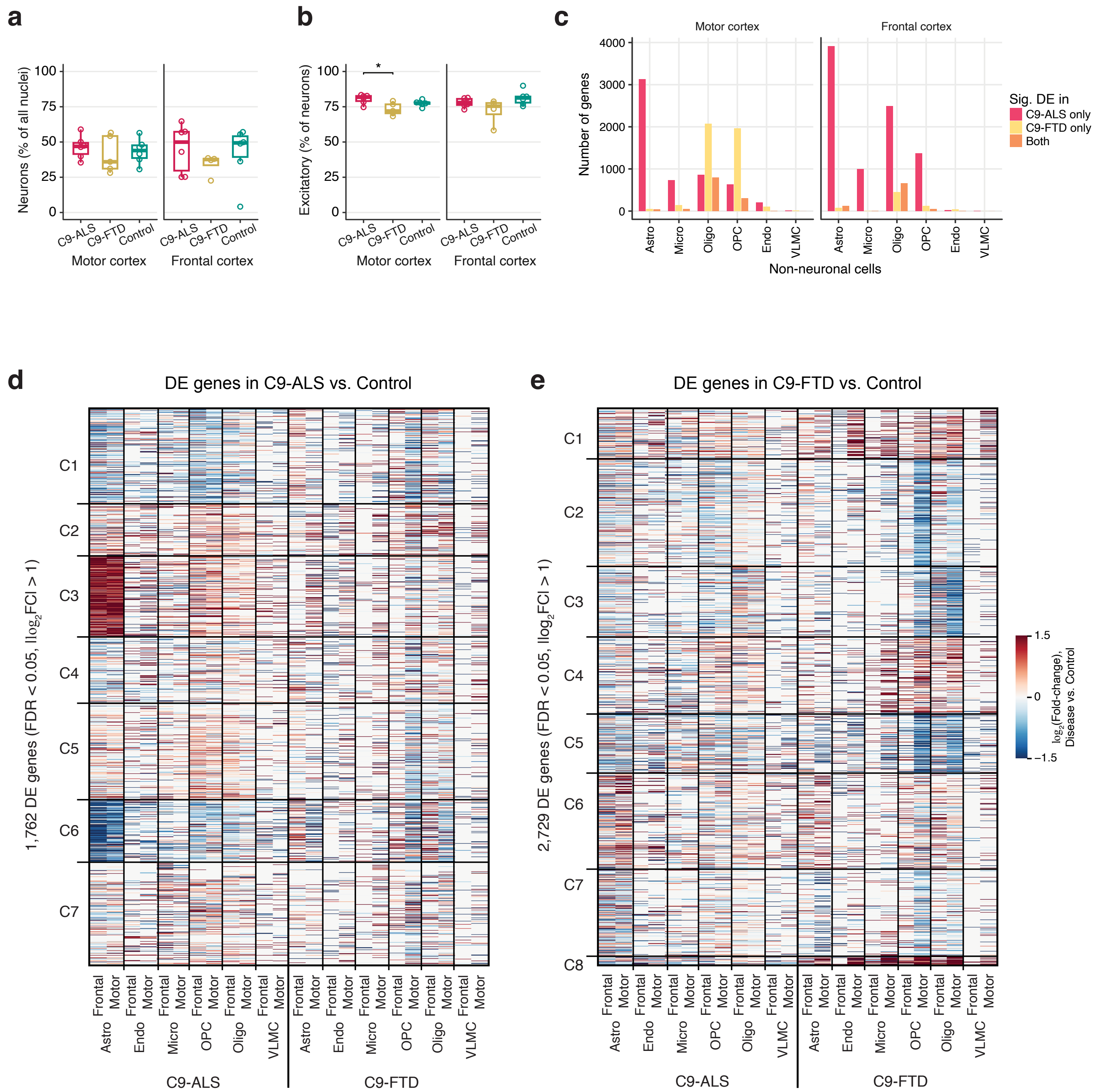

Supplementary Fig. 12. Comparison of the disease effect on gene expression in non-neuronal cells in C9-ALS and C9-FTD. (a-b) Related to Fig. 6b-c. Relative abundance of neurons (a), and percent of excitatory neurons among all neurons (b) using relaxed QC criteria (without thresholds on the number of detected genes, the percentage of mitochondrial reads, and doublet scores). This analysis included all 195,144 non-empty droplets determined by CellBender[7]. No significant difference in the percentage of neurons was detected between diagnostic groups in (a) (two-sided Welch's t-test, p=0.20-0.76). Circles represent individual donors; N = 6 C9-ALS and 6 control samples for both brain regions, and N = 5 and 4 C9-FTD samples from motor cortex and frontal cortex, respectively. *, two-sided Welch's t-test p = 0.021. In each box plot, the lower and upper hinges correspond to the first and third quartiles; the whiskers extend 1.5 * IQR (interquartile range) away from the hinges; and the center denotes the median. (c) Number of significant DE genes identified in the two diseases. For this analysis, control samples were split into two independent groups (from different donors) for

comparison with C9-ALS and with C9-FTD to avoid double dipping (see Methods). (d-e) Heatmap to compare the gene expression fold-changes of C9-ALS vs. control and C9-FTD vs. control. Strongly DE genes (fold-change > 2) identified from the full dataset (same genes shown in Fig. 2d for C9-ALS and Fig. 6h for C9-FTD) were used, and the fold-changes from the split control analysis were shown to avoid double dipping. Source data are provided as a Source Data file.

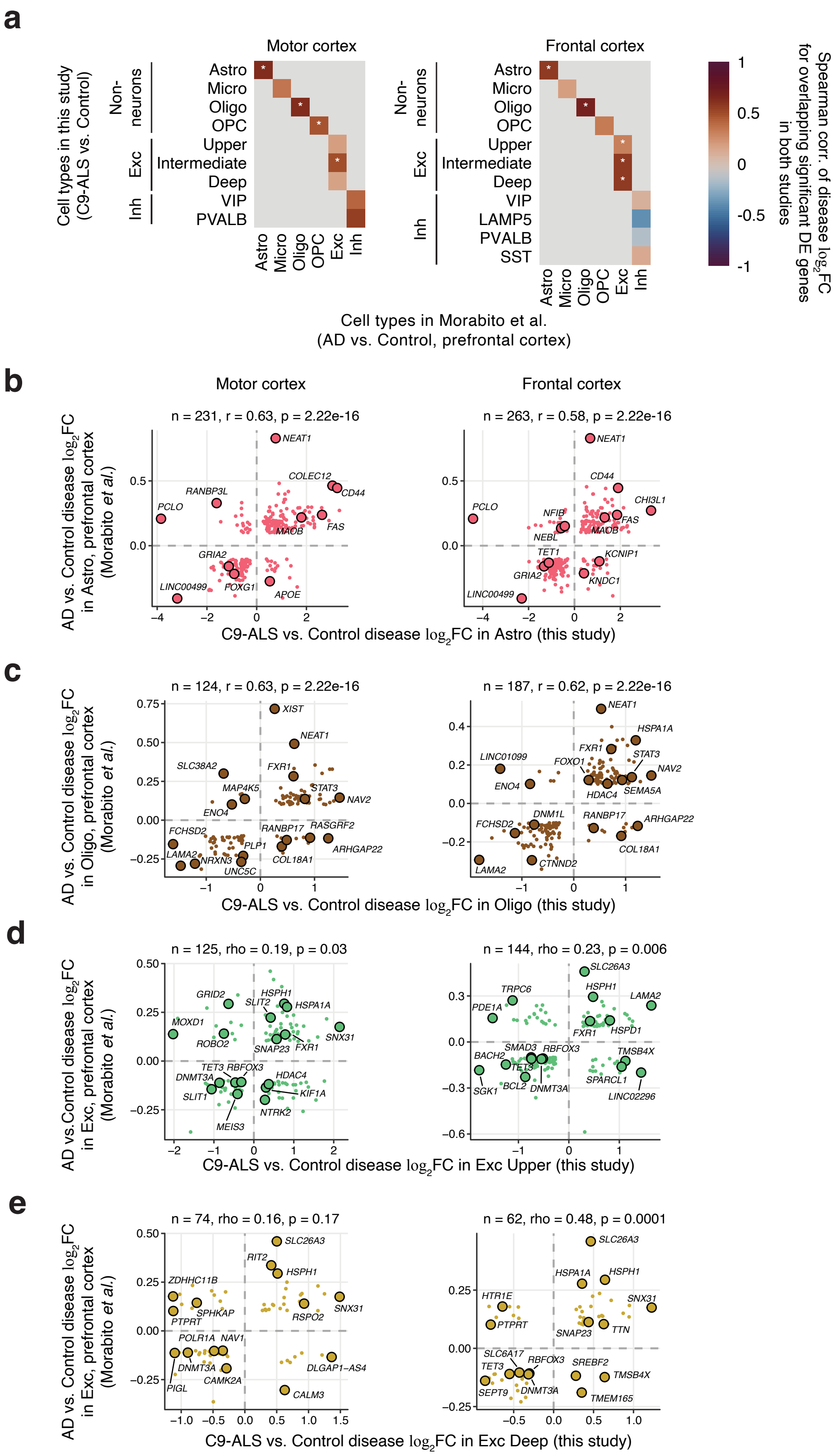

Supplementary Fig. 13. Comparison of C9-ALS vs. control gene expression fold-changes with AD vs. control gene expression fold-changes. (a) Spearman correlations of disease fold-changes between C9-ALS (this study) and AD[8] for matching cell types. Only genes that were significantly DE in both diseases were included in this analysis. Asterisk denotes significant correlations (FDR < 0.05). (b-e) Scatter plots showing the

correlation of the disease fold-changes between the two diseases for astrocytes, oligodendrocytes, and excitatory neurons in the two studies. Examples of genes with concordant or discordant changes in the two diseases are highlighted. n, number of significant DE genes in both studies in the corresponding cell types; rho and p, two-sided Spearman's rank correlation test coefficient and p-value. Source data are provided as a Source Data file.

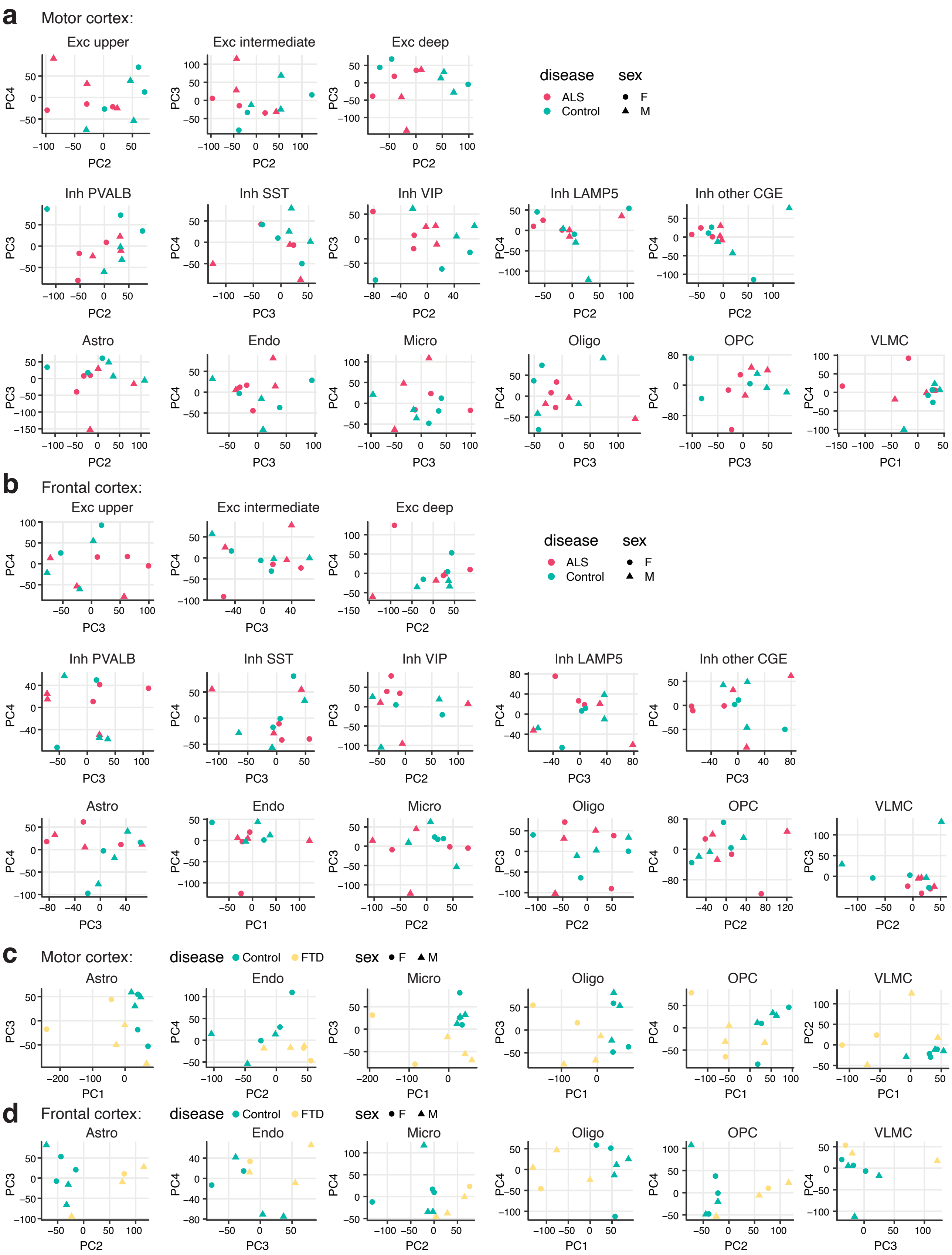

Supplementary Fig. 14. Principal component analysis of the pseudobulk snRNA-seq counts from each donor in each cell type. (a-b) Separation of the pseudobulk gene expression profiles in each major cell type

for C9-ALS and control donors in motor cortex (a) and frontal cortex (b). (c-d) Separation of the pseudobulk gene expression profiles in each non-neuronal nuclei for C9- FTD and control donors in motor cortex (c) and frontal cortex (d). Counts were normalized with DEseq2's median of ratios method[9], and the natural logarithm of the normalized count plus one was used in the principal component analysis. CGE, caudal ganglionic eminence; Astro, astrocytes; Endo, endothelial cells; Micro, microglia; Oligo, oligodendrocytes; OPC, oligodendrocyte precursor cells; VLMC, vascular leptomeningeal cell; Exc, excitatory neurons; Inh, inhibitory neurons; PC, principal component. Source data are provided as a Source Data file.

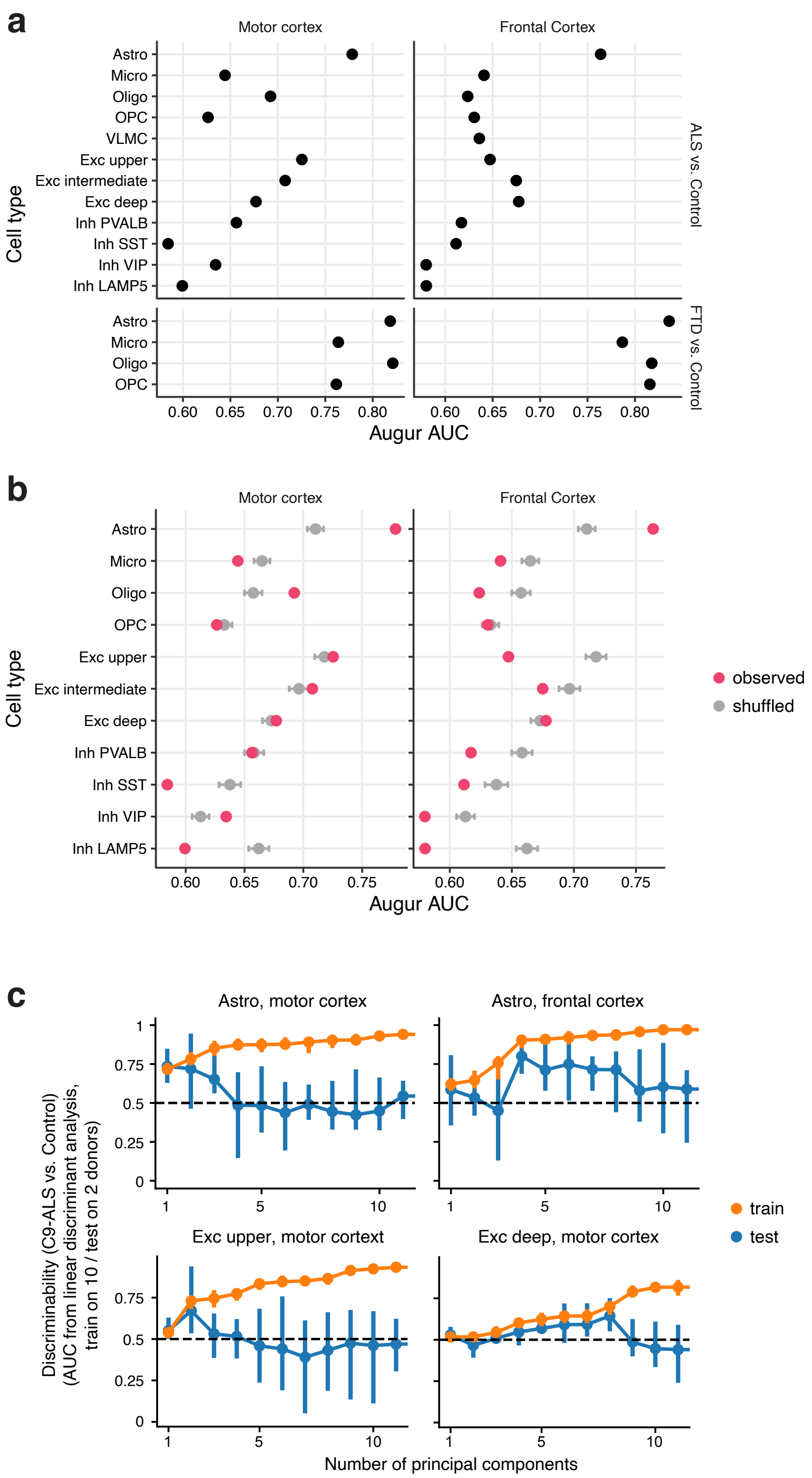

Supplementary Fig. 15. Separations of disease and control samples in the snRNA-seq dataset. (a-b) Cell type prioritization using Augur[10]. (a) The area under the receiver operating characteristic curve (AUC) was reported by Augur as a metric of the separability between the disease (C9-ALS or C9-FTD) and control samples. (b) For C9-ALS vs. control, n = 82 random shuffles with balanced diagnosis and sex were generated by permutation of the diagnosis labels of the donors. Augur was run on these shuffles and the mean AUCs are shown in grey dots. Data are presented as mean values +/- 95% confidence intervals of the mean. Observed

AUCs with real diagnosis labels are shown in red dots. Cell types that were not detected in all 12 donors were omitted in this analysis. (c) Discriminability of C9-ALS vs. control cells is shown as the area under the receiver operating characteristic (ROC) for four sets of cells. Donors were split using k-fold cross-validation, with 10 subjects used for training a linear discriminant classifier and 2 subjects used for testing (one control and one C9-ALS subject). The AUC for training subjects (orange) increases with the number of PCs used, whereas the test-set AUC (blue) shows that the C9-ALS cells can be significantly discriminated from the control cells using the top few PCs. Data are presented as mean values +/- 95% confidence intervals of the mean; n = 6 groups of split samples. Astro, astrocytes; Endo, endothelial cells; Micro, microglia; Oligo, oligodendrocytes; OPC, oligodendrocyte precursor cells; VLMC, vascular leptomeningeal cell; Exc, excitatory neurons; Inh, inhibitory neurons; AUC, area under the ROC curve. Source data are provided as a Source Data file.

Supplementary References

1. Wolock, S. L., Lopez, R. & Klein, A. M. Scrublet: Computational Identification of Cell Doublets in Single-Cell Transcriptomic Data. *Cell Syst* **8**, 281–291.e9 (2019).
2. McInnes, L., Healy, J. & Melville, J. UMAP: Uniform Manifold Approximation and Projection for Dimension Reduction. *arXiv [stat.ML]* (2018).
3. Zappia, L. & Oshlack, A. Clustering trees: a visualization for evaluating clusterings at multiple resolutions. *Gigascience* **7**, (2018).
4. Kiselev, V. Y. *et al.* SC3: consensus clustering of single-cell RNA-seq data. *Nat. Methods* **14**, 483–486 (2017).
5. Bakken, T. E. *et al.* Comparative cellular analysis of motor cortex in human, marmoset and mouse. *Nature* **598**, 111–119 (2021).
6. Schep, A. N., Wu, B., Buenrostro, J. D. & Greenleaf, W. J. chromVAR: inferring transcription-factor-associated accessibility from single-cell epigenomic data. *Nat. Methods* **14**, 975–978 (2017).
7. Fleming, S. J., Marioni, J. C. & Babadi, M. CellBender remove-background: a deep generative model for unsupervised removal of background noise from scRNA-seq datasets. *bioRxiv* 791699 (2019) doi:10.1101/791699.
8. Morabito, S. *et al.* Single-nucleus chromatin accessibility and transcriptomic characterization of Alzheimer's disease. *Nat. Genet.* **53**, 1143–1155 (2021).
9. Love, M. I., Huber, W. & Anders, S. Moderated estimation of fold change and dispersion for RNA-seq data with DESeq2. *Genome Biol.* **15**, 550 (2014).
10. Skinnider, M. A. *et al.* Cell type prioritization in single-cell data. *Nat. Biotechnol.* (2020) doi:10.1038/s41587-020-0605-1.
