# Supplementary material for: Divergent single cell transcriptome and epigenome alterations in ALS and FTD patients with C9orf72 mutation
Source: Nat Commun. 2023 Sep 15;14:5714. doi: 10.1038/s41467-023-41033-y (PMC10504300; doi:10.1038/s41467-023-41033-y)
Supplement: Supplementary file 3 — Description of additional supplementary files [file 41467_2023_41033_MOESM3_ESM.pdf]

Description of Additional Supplementary Files Document

Supplementary Dataset 1. Metadata of the samples used in this study.

Supplementary Dataset 2. Metadata and cell type annotations of all high-quality nuclei in our snRNA-seq dataset.

Supplementary Dataset 3. List of C9-ALS vs. control differentially expressed genes identified in each major cell type in motor cortex.

Supplementary Dataset 4. List of C9-ALS vs. control differentially expressed genes identified in each major cell type in frontal cortex.

Supplementary Dataset 5. Gene Ontology enrichment results for C9-ALS vs. control differentially expressed genes.

Supplementary Dataset 6. Metadata and cell type annotations of all high-quality nuclei in our snATAC-seq dataset.

Supplementary Dataset 7. H3K27ac differential peaks between C9-ALS and control identified in ChIP-seq.

Supplementary Dataset 8. List of C9-FTD vs. control differentially expressed genes identified

in each non-neuronal cell type in motor cortex.

Supplementary Dataset 9. List of C9-FTD vs. control differentially expressed genes identified in each non-neuronal cell type in frontal cortex.

Supplementary Dataset 10. Digital Western Blot signal for tested genes in C9-ALS and control samples.

Supplementary Dataset 11. Correlations of snATAC-seq and H3K27ac ChIP-seq signal in 1kb genomic bins in C9-ALS and control samples.

Supplementary Dataset 12. Spearman correlation of the C9-ALS vs. control fold-changes for snRNA expression vs. snATAC gene activity score.

Supplementary Dataset 13. Bulk RNA-seq seq differential expression analysis results using edgeR.
