# Supplementary material for: Divergent single cell transcriptome and epigenome alterations in ALS and FTD patients with C9orf72 mutation
Source: Nat Commun. 2023 Sep 15;14:5714. doi: 10.1038/s41467-023-41033-y (PMC10504300; doi:10.1038/s41467-023-41033-y)
Supplement: Supplementary file 17 — Reporting Summary [file 41467_2023_41033_MOESM17_ESM.pdf]

Corresponding author(s): Stella Dracheva and Eran Mukamel

Last updated by author(s): Aug 11, 2023

Reporting Summary

Nature Portfolio wishes to improve the reproducibility of the work that we publish. This form provides structure for consistency and transparency in reporting. For further information on Nature Portfolio policies, see our Editorial Policies and the Editorial Policy Checklist.

Statistics

For all statistical analyses, confirm that the following items are present in the figure legend, table legend, main text, or Methods section.

n/a	Confirmed	
☐	☒	The exact sample size (*n*) for each experimental group/condition, given as a discrete number and unit of measurement
☐	☒	A statement on whether measurements were taken from distinct samples or whether the same sample was measured repeatedly
☐	☒	The statistical test(s) used AND whether they are one- or two-sided<br>*Only common tests should be described solely by name; describe more complex techniques in the Methods section.*
☐	☒	A description of all covariates tested
☐	☒	A description of any assumptions or corrections, such as tests of normality and adjustment for multiple comparisons
☐	☒	A full description of the statistical parameters including central tendency (e.g. means) or other basic estimates (e.g. regression coefficient) AND variation (e.g. standard deviation) or associated estimates of uncertainty (e.g. confidence intervals)
☐	☒	For null hypothesis testing, the test statistic (e.g. *F*, *t*, *r*) with confidence intervals, effect sizes, degrees of freedom and *P* value noted<br>*Give P values as exact values whenever suitable.*
☒	☐	For Bayesian analysis, information on the choice of priors and Markov chain Monte Carlo settings
☐	☒	For hierarchical and complex designs, identification of the appropriate level for tests and full reporting of outcomes
☐	☒	Estimates of effect sizes (e.g. Cohen's *d*, Pearson's *r*), indicating how they were calculated

Our web collection on statistics for biologists contains articles on many of the points above.

Software and code

Policy information about availability of computer code

Data collection

No software was used for data collection.

Data analysis

Codes used in this study are available at https://github.com/hoholee/C9_ALS_FTD_single_nuclei_transcriptome_epigenome.
The following are individual packages used in the analysis in this study:

snRNA-seq read mapping: 10x Genomics Cell Ranger (v3.0.2)
snRNA-seq cell-free droplets and background noise removal: CellBender (v0.2.0)
Doublet removal: Scrublet (v0.2.3)
snRNA-seq data processing: Seurat (v4.0.4)
Multimodal data integration: Harmony (v0.1.0)
Evaluate and visualize the stability of the snRNA-seq clusters: Clustree (v0.5.0)
Cell type prioritization: Augur (v1.0.3)
snRNA-seq differential expression test: MAST (v1.18.0) (https://github.com/RGLab/MAST)
Gene Ontology enrichment test: WebGestalt (http://www.webgestalt.org/, v2019)
Sequencing read quality control: FastQC (v0.11.8)
Adapter trimming: Trim Galore (v0.5.0), Cutadapt (v1.16)
Bulk RNA-seq read mapping: STAR (Spliced Transcripts Alignment to a Reference, v 2.7.1a)
Bulk RNA-seq read counting: RSEM (RNA-Seq by Expectation Maximization, v1.2.30)
Bulk RNA-seq differential expression test: edgeR (v3.34.1); DEseq2 (v1.36.0)
snATAC-seq read mapping: 10x Genomics Cell Ranger ATAC (v1.1.0)
snATAC-seq barcode multiplets removal: “clean_barcode_multiplets_1.1.py” script (provided by 10x Genomics)

snATAC-seq processing: ArchR (v1.0.2)
Peak calling: MACS2 (v2.2.7.1)
Motif sequence logo creation: MEME Suite (v5.4.1)
ChIP-seq data preprocessing: HTStream tool (https://github.com/s4hts/HTStream)
ChIP-seq read mapping: BWA-MEM2
SAM and BAM file manipulation: Samtools (v1.9)
Genomic regions manipulation: BEDTools (v2.27.1)
ChIP-seq signal computation: deepTools (v.3.3.1)
ChIP-seq differential test: DiffBind (v3.2.7)
General scripting and data visualization: R (4.1.2), Python (v 3.7.12)

For manuscripts utilizing custom algorithms or software that are central to the research but not yet described in published literature, software must be made available to editors and reviewers. We strongly encourage code deposition in a community repository (e.g. GitHub). See the Nature Portfolio guidelines for submitting code & software for further information.

Data

Policy information about availability of data

All manuscripts must include a data availability statement. This statement should provide the following information, where applicable:

- Accession codes, unique identifiers, or web links for publicly available datasets
- A description of any restrictions on data availability
- For clinical datasets or third party data, please ensure that the statement adheres to our policy

All sequencing datasets generated in this study have been deposited in the Gene Expression Omnibus repository with the accession number GSE219281.

The following are publicly available genomic dataset used in this study:

Homo Sapiens reference genome: hg38 version downloaded from iGenome (https://support.illumina.com/sequencing/sequencing_software/igenome.html)
Gene annotation files: GENCODE release V35 (https://www.gencodegenes.org/human/releases.html)
The non-redundant transcription factor (TF) motif archetypes (v2.0-beta, https://github.com/jvierstra/motif-clustering)

Human research participants

Policy information about studies involving human research participants and Sex and Gender in Research.

Reporting on sex and gender	The study analyzed human postmortem brain tissues from donors with C9-ALS (N=11), C9-FTD (N=7), and controls who were pathologically normal (N=15) (see Supplementary Dataset 1). Both male (a total of N=17) and female (a total of N=16) subjects were represented in the cohorts that were used for snRNA/ATAC-seq and FANS RNA/ChIP-seq assays, and data were collected for both sexes. However, sex-based analyses were not performed, because in each assay, small samples sizes did not allow us to perform statistically meaningful comparisons.
Population characteristics	The following population characteristics were considered in the study: sex, age, disease status (ALS, FTD, or pathologically normal), and the presence of C9orf72 mutation. Population characteristics is provided in Supplementary Dataset 1.
Recruitment	NA (This is a postmorterm study).
Ethics oversight	The tissues were obtained from the Mayo Clinic Brain Bank (Jacksonville, FL USA). Analysis was conducted as exempt human research, considering that these were postmortem brain samples, and they were not specifically collected for this study.

Note that full information on the approval of the study protocol must also be provided in the manuscript.

Field-specific reporting

Please select the one below that is the best fit for your research. If you are not sure, read the appropriate sections before making your selection.

☒ Life sciences ☐ Behavioural & social sciences ☐ Ecological, evolutionary & environmental sciences

For a reference copy of the document with all sections, see nature.com/documents/nr-reporting-summary-flat.pdf

Life sciences study design

All studies must disclose on these points even when the disclosure is negative.

Sample size	No statistical methods were used to predetermine sample size because effect sizes were unknown before experiments. Samples sizes were chosen based on the availability of ALS/FTD samples with C9-orf72 (C9) mutation.
Data exclusions	No samples were excluded from analysis. For each single cell transcriptome or ATAC-seq data set we applied quality control (QC) criteria to exclude single cells from downstream analyses based on criteria including the total number of sequenced reads, and mitochondrial RNA fraction.

Replication

For our studies on specific cell types, it's necessary to isolate nuclei prior to conducting experiments. This process requires a significant volume of brain tissue. Given the limited availability of postmortem tissue from donors with C9-ALS and C9-FTD, we were unable to use a separate replication cohort for these studies. As a result, we only performed one technical replicate for each donor in our snRNA-seq, snATAC-seq, bulk cell-type-specific RNA-seq, and bulk cell-type-specific ChIP-seq experiments. We used fluorescence-activated nuclear sorting (FANS) to isolate bulk cell-type-specific nuclei.

We verified the reproducibility of analyses by using samples from multiple independent disease and control donors (N=17 donors for snRNA-seq and snATAC-seq, 20 donors for bulk cell-type-specific RNA-seq, and 15 donors for bulk cell-type-specific ChIP-seq). Each of these analyses considered biological replicates, defining a false discovery rate that was calibrated by the variability across donors.

In addition, we used two complementary assays to determine the epigenetic landscape of C9-ALS brain cells, snATAC-seq, and bulk cell-type-specific H3K27ac ChIP-seq in FANS-separated major brain cell types. The ChIP-seq data correlated strongly with the snATAC-seq tracks from corresponding cell types.

Lastly, we connected transcriptional alterations in C9-ALS to their protein expression using automated Western blotting. Antibodies against 19 differentially expressed (DE) gene products detected a protein band of the expected size in control samples. Using these antibodies, we confirmed the statistical significance of the predicted changes in protein levels for 6 of the 19 DE genes, with no discordant changes observed for any of the tested proteins.

We conducted automated Western blotting using tissue homogenates without isolating nuclei beforehand. This approach required less tissue, enabling us to assess two technical replicates per donor and gene target. We used the average signals from the two replicates for each donor and target as our observations. Comparisons yielding a p-value less than 0.05 were considered statistically significant.

Randomization

To avoid batch effects, nuclear isolations and library preparations for snRNA-seq, snATAC-seq, FANS RNA-seq, and FANS ChIP-seq were randomized between diagnoses (ALS/FTD and control).

No randomization was required for Western blotting experiments, as all available C9-ALS and control samples were assessed in one experiment for each target protein.

Blinding

The technical personnel who performed the nuclear isolations and library preparations was not aware of the sample diagnosis. Case control analyses were performed in the study and could not be achieved blindly.

Reporting for specific materials, systems and methods

We require information from authors about some types of materials, experimental systems and methods used in many studies. Here, indicate whether each material, system or method listed is relevant to your study. If you are not sure if a list item applies to your research, read the appropriate section before selecting a response.

Materials & experimental systems

n/a	Involved in the study
☐	☒ Antibodies
☒	☐ Eukaryotic cell lines
☒	☐ Palaeontology and archaeology
☒	☐ Animals and other organisms
☒	☐ Clinical data
☒	☐ Dual use research of concern

Methods

n/a	Involved in the study
☐	☒ ChIP-seq
☐	☒ Flow cytometry
☒	☐ MRI-based neuroimaging

Antibodies

Antibodies used

Antibodies List. Only commercial antibodies were used in this paper.

1. PE-conjugated anti-NeuN (Millipore, Cat. # FCMAB317PE) https://www.emdmillipore.com/US/en/product/Milli-Mark-Anti-NeuN-PE-Antibody-clone-A60,MM_NF-FCMAB317PE
2. Custom-conjugated to Alexa647 anti-SOX10 (R&D Systems, Cat. # AF2864) https://www.rndsystems.com/products/human-sox10-antibody_af2864
3. Alexa488-conjugated anti-IRF5 (R&D Systems, Cat. # IC4508G) https://www.rndsystems.com/products/human-irf5-alexa-fluor-488-conjugated-antibody_ic4508g
4. Anti-GFAP (Millipore Sigma, Cat. #AB5804) https://www.sigmaaldrich.com/US/en/product/mm/ab5804
5. Anti-HSP90 (R&D Systems Cat, # MAB3286) https://www.rndsystems.com/products/human-mouse-rat-hsp90-antibody-341320_mab3286
6. Anti-GFAP (Sigma Cat. # G3893) https://www.sigmaaldrich.com/US/en/product/sigma/g3893?gclid=Cj0KCQiA14WdBhD8ARIsANao07hNT_zgM_8IdsIUNEqq622q-Jay-BeaA7HdeOVmQDUGFVBFi1CBJfEaAs_SEALw_wcB&gclsrc=aw.ds
7. Anti-CD44 (R&D Systems, Cat. # BBA10) https://www.rndsystems.com/products/human-cd44s-pan-specific-antibody-2c5_bba10
8. Anti-CHI3L1 (R&D Systems, Cat. # AF2599) https://www.rndsystems.com/products/human-chitinase-3-like-1-antibody_af2599
9. Anti-HSP70 (R&D Systems, Cat. # AF1663) https://www.rndsystems.com/products/human-mouse-rat-hsp70-hspa1a-antibody_af1663

10. Anti-HSP27 (R&D Systems, Cat # AF1580) https://www.rndsystems.com/products/human-mouse-hsp27-antibody_af1580
11. Anti-RANBP3L (Novus Cat # NBP2-38347) https://www.novusbio.com/products/ranbp3l-antibody_nbp2-38347
12. Anti-KCND3/Kv4.3 (Novus Cat. # NBP2-76945) https://www.novusbio.com/products/kv43-antibody_nbp2-76945
13. Anti-DNMT3A (Novus Cat # 64B1446) https://www.novusbio.com/search?keywords=64B1446
14. Anti-NEFL (R&D Cat # MAB22163; Clone #1002615) https://www.novusbio.com/products/nf-l-antibody-1002615_mab22163
15. Anti-Clusterin (Novus Cat. # MAB2937) https://www.novusbio.com/products/clusterin-apoj-antibody-350227_mab2937
16. Anti-RAP1GAP (Novus Cat. # NBP1-53072) https://www.novusbio.com/products/rap1gap-antibody_nbp1-53072
17. Anti-Ubiquitin B (Novus Cat. # NBP3-07163) https://www.novusbio.com/products/ubiquitin-b-antibody-ubb-1748_nbp3-07163
18. Anti-TGFB2 (Novus Cat # AB-112-NA) https://www.novusbio.com/products/tgf-beta-2-antibody_ab-112-na
19. Anti-SOD1 (R&D Systems; Cat # AF3418) https://www.novusbio.com/products/sod1-cu-zn-sod-antibody_af3418
20. Anti-H3K27ac (Active Motif Cat# 39133) https://www.activemotif.com/catalog/details/39133/histone-h3-acetyl-lys27-antibody-pab
21. Anti-Monoamine Oxidase B Antibody (5M8A5) (Novus Cat # NBP3-15411) https://www.novusbio.com/primary-antibodies/monoamine-oxidase-b
22. Anti-ATP5A Antibody, (Novus Cat # NBP2-92928) https://www.novusbio.com/products/atp5a-antibody_nbp2-92928
23. Anti-Cytochrome c Antibody (Novus Cat #NBP2-21569) https://www.novusbio.com/products/cytochrome-c-antibody_nbp2-21569
24. Anti- COX5A Antibody (Novus, Cat # NBP1-32550; 1:10) https://www.novusbio.com/products/cox5a-antibody_nbp1-32550

Validation

Antibodies that were used in different assays.

Fluorescence-activated nuclear sorting (FANS).
1. PE-conjugated anti-NeuN (Millipore, Cat. # FCMAB317PE), Clone A60; species reactivity-human; host-mouse; type-monoclonal antibody.
Description: The antibodies recognize the DNA-binding, neuron-specific protein NeuN, which is present in most CNS and PNS neuronal cell types of all vertebrates tested. NeuN protein distributions are restricted to neuronal nuclei, perikarya and some proximal neuronal processes in both fetal and adult brain although, some neurons fail to be recognized by NeuN at all age (e.g., Cajal-Retzius cells, Purkinje cells, inferior olivary and dentate nucleus neurons, and sympathetic ganglion cells) (Mullen et al., 1992; Wolf et al., 1996).

2. Custom-conjugated to Alexa647 anti-SOX10 (R&D Systems, Cat. # AF2864) species reactivity-human; host-goat; type-polyclonal goat IgG.
Description: The antibodies recognize SOX10, which belongs to the SOX family of transcription factors and is a pan-oligodendrocyte lineage marker. These antibodies were validated in numerous human cell lines (company website) as well as in human postmortem brain tissue (Ernst et al., Cell, 2014).

3. Alexa488-conjugated anti-IRF5 (R&D Systems, Cat. # IC4508G); species reactivity-human; host-sheep; type- polyclonal sheep IgG.
Description: The antibodies recognize IRF5. IRF5 is a member of the IRF family of transcription factors that show diverse cellular regulation of interferon-stimulated gene transcription and are highly enriched in myeloid cells, including CNS microglia (Zhang et al. Neuron, 2016). Antibodies against IRF8 were previously validated for FANS-isolation of microglia nuclei from human postmortem brain (van der Poel et. al., Nature Communications, 2019). We validated the IC4508G anti-IRF5 antibodies for FANS-isolation of microglia nuclei from human postmortem cells in this paper (Extended Data Fig. 5).

GFAP immunofluorescence
GFAP primary antibodies (Millipore Sigma, Cat. #AB5804); species reactivity-human, bovine, canine, rat; host- rabbit; type-polyclonal rabbit.
Description: The antibodies recognize glial fibrillary acidic protein (GFAP), which labels brain astrocytes and Bergmann glia cells.

Western blot assay
1. Anti-HSP90 (R&D Systems Cat, # MAB3286), Clone #341320; species reactivity-human, mouse, rat; host-mouse; type-monoclonal mouse IgG2B.
Description: HSP90 is a composite name for a large group of Heat Shock Proteins whose molecular weights average 90 kDa. Eukaryotic cells have two principal isoforms of HSP90. These antibodies react with the 724 amino acid constitutively expressed HSP90AB1-isoform 1 (also known as HSP90 beta, HSP90B, HSPCB, HSPC2, and HSP89 beta) as well as with the 737 amino acid inducible form, HSP90AA1 (also known as HSP90 alpha, HSP90A, HSPC1, HSPCA, HSP89 alpha and LAP2).

2. Anti-GFAP (Sigma Cat, # G3893), Clone G-A-5; species reactivity-human, rat, pig; host-mouse; type-monoclonal mouse IgG1.
Description: The antibodies recognize glial fibrillary acidic protein (GFAP), which labels brain astrocytes and Bergmann glia cells.

3. Anti-CD44 (R&D Systems, Cat. # BBA10), Clone # 2C5; species reactivity-human; host-mouse; type-monoclonal mouse IgG2A.
Description: The antibodies recognize a non-kinase transmembrane proteoglycan CD44. CD44 is a ubiquitously expressed protein and is expressed in activated astrocytes (Sosunov et al. J Neurosci. 2015).

4. Anti-CHI3L1 (R&D Systems, Cat. # AF2599); species reactivity-human; host-goat; type-polyclonal goat IgG.
Description: The antibodies recognize CHI3L1, also known as HCgp39. CHI3L1is a secreted glycoprotein that belongs to the family of chitinase-like lectins. Elevated levels of chitinases (including CHI3L1) have been proposed as a biomarker of ALS severity and progression (Gaur et al. Front. Neurol. 2020).

5. Anti-HSP70 (R&D Systems, Cat. # AF1663); species reactivity-human, mouse, rat; host-rabbit; type-polyclonal rabbit IgG.
Description: The antibodies recognize HSP70, which is a 72 kDa member of the heat shock protein 70 family of proteins. HSP70 is also known as HSPA1A, HSP70-1, and HSP72.

6. Anti-HSP27 (R&D Systems, Cat # AF1580); species reactivity-human, mouse; host-rabbit; type-polyclonal rabbit IgG.
Description: The antibodies recognize HSP27, also known as HSPB1, which is a member of the small heat shock protein family.

7. Anti-RANBP3L (Novus Cat # NBP2-38347); species reactivity-human; host-rabbit; type-polyclonal rabbit IgG.
Description: The antibodies recognize RANBP3L protein, which is a nuclear export factor.

8. Anti-KCND3/Kv4.3 (Novus Cat. # NBP2-76945); species reactivity-human, mouse; host-rabbit; type-polyclonal rabbit IgG.
Description: The antibodies recognize KCND3 protein (both KCND3L and KCND3S isoforms), also known as Kv4.3 channel. Kv4.3 is a potassium voltage-gated channel subfamily D member 3.

9. Anti-DNMT3A (Novus Cat # 64B1446), Clone # 2C5; species reactivity-human, mouse, rat; host-mouse; type-monoclonal mouse IgG1.
Description: The antibodies recognize DNA (cytosine-5-)-methyltransferase 3 alpha (DNMT3A), which is involved in de novo cytosine methylation of genomic DNA.

10. Anti-NEFL (R&D Cat # MAB22163), Clone # 64B1446; species reactivity-human, mouse, rat; host-mouse; type-monoclonal mouse IgG2B.
Description: The antibodies recognize is a 68 kDa light chain cytoskeletal intermediate filament protein (NF-L) that is expressed in neurons.

11. Anti-Clusterin (Novus Cat. # MAB2937), Clone # 350227; species reactivity-human; host-mouse; type-monoclonal mouse IgG2B.
Description: The antibodies recognize clusterin (CLU), also known as Apolipoprotein J, Sulfated Glycoprotein 2 (SGP-2), TRPM-2, or SP-40. Clusterin is a secreted multi-functional protein that function as a chaperone of misfolded extracellular proteins.

12. Anti-RAP1GAP (Novus Cat. # NBP1-53072); species reactivity-human; host-rabbit; type-polyclonal rabbit IgG.
Description: The antibodies recognize RAP1GAP protein, which is the GTPase activator for the nuclear Ras-related regulatory protein RAP-1A.

13. Anti-UbiquitinB (Novus Cat. # NBP3-07163), Clone # UBB/1748; species reactivity-human; host-mouse; type-monoclonal mouse IgG2c.
Description: The antibodies recognize ubiquitin. Ubiquitin is covalently bound to proteins to be degraded, labeling these proteins for degradation.

14. Anti-TGFB2 (Novus Cat # AB-112-NA); species reactivity-All-Multi; host-goat; type-polyclonal goat IgG.
Description: The antibodies recognize TGF-beta 2 (transforming growth factor beta 2) protein, which is a cytokine that acts as cellular switch that regulates multiple processes (e.g., immune function and proliferation).

15. Anti-SOD1 (R&D Systems; Cat # AF3418); species reactivity-human, mouse; host-goat; type-polyclonal goat IgG.
Description: The antibodies recognize SOD1, also known as Cu/Zn SOD or soluble SOD. SOD1 is responsible for destroying free superoxide radicals in the body. It is a soluble, cytoplasmic 16 kDa homodimer,

16. Anti-Monoamine Oxidase B Antibody (5M8A5) (Novus; Cat # NBP3-15411); species reactivity-Hu, Mu, Rt; host-rabbit; type-monoclonal.
Description: The antibodies recognize monoamine oxidase B protein, which is an enzyme located in the mitochondrial outer membrane. It catalyzes the oxidative deamination of biogenic and xenobiotic amines.

17. Anti-ATP5A Antibody, (Novus; Cat NBP2-92928); species reactivity-Hu, Mu, Rt; host-rabbit; type-polyclonal.
Description: The antibodies recognize ATP5A that encodes a subunit of mitochondrial ATP synthase.

18. Anti-Cytochrome c Antibody (Novus; Cat #NBP2-21569); species reactivity-Hu, Mu, Rt, Po, Bv; host-rabbit; type-polyclonal.
Description: The antibodies recognize cytochrome c, a component of the electron transport chain in mitochondria.

19. Anti- COX5A Antibody (Novus, Cat # NBP1-32550; 1:10); species reactivity-Hu, Mu, Ze, RtPo, Bv; Ca, Pm, Rm; host-rabbit; type-polyclonal.
Description: The antibodies recognize a subunit of cytochrome c oxidase (COX), which is the terminal enzyme of the mitochondrial respiratory chain

ChIP-seq
Anti-H3K27ac (Active Motif Cat# 39133); species reactivity-human; host-rabbit; type-polyclonal rabbit IgG.
Description: The antibodies recognize acetylated lysine 27 of histone 3, which is the marker of active promoters and enhancers. The antibodies were previously validated for ChIP-seq and have been used in numerous studies.

ChIP-seq

Data deposition

☒ Confirm that both raw and final processed data have been deposited in a public database such as GEO.

☒ Confirm that you have deposited or provided access to graph files (e.g. BED files) for the called peaks.

Data access links
May remain private before publication.

https://www.ncbi.nlm.nih.gov/geo/query/acc.cgi?acc=GSE219277
Password: wdubiisotfcjfqt

Files in database submission

ChIPseq_C9ALS_A10_Astrocytes_H3K27ac_L001_R1.fastq.gz
ChIPseq_C9ALS_A10_Astrocytes_H3K27ac_L001_R2.fastq.gz
ChIPseq_C9ALS_A10_Astrocytes_H3K27ac_L004_R1.fastq.gz
ChIPseq_C9ALS_A10_Astrocytes_H3K27ac_L004_R2.fastq.gz
ChIPseq_C9ALS_A10_Microglia_H3K27ac_L001_R1.fastq.gz
ChIPseq_C9ALS_A10_Microglia_H3K27ac_L001_R2.fastq.gz
ChIPseq_C9ALS_A10_Microglia_H3K27ac_L004_R1.fastq.gz
ChIPseq_C9ALS_A10_Microglia_H3K27ac_L004_R2.fastq.gz
ChIPseq_C9ALS_A10_Neurons_H3K27ac_L001_R1.fastq.gz
ChIPseq_C9ALS_A10_Neurons_H3K27ac_L001_R2.fastq.gz
ChIPseq_C9ALS_A10_Neurons_H3K27ac_L004_R1.fastq.gz
ChIPseq_C9ALS_A10_Neurons_H3K27ac_L004_R2.fastq.gz
ChIPseq_C9ALS_A10_OligoLineage_H3K27ac_L001_R1.fastq.gz
ChIPseq_C9ALS_A10_OligoLineage_H3K27ac_L001_R2.fastq.gz
ChIPseq_C9ALS_A10_OligoLineage_H3K27ac_L004_R1.fastq.gz
ChIPseq_C9ALS_A10_OligoLineage_H3K27ac_L004_R2.fastq.gz
ChIPseq_C9ALS_A11_Astrocytes_H3K27ac_L001_R1.fastq.gz
ChIPseq_C9ALS_A11_Astrocytes_H3K27ac_L001_R2.fastq.gz
ChIPseq_C9ALS_A11_Astrocytes_H3K27ac_L004_R1.fastq.gz
ChIPseq_C9ALS_A11_Astrocytes_H3K27ac_L004_R2.fastq.gz
ChIPseq_C9ALS_A11_Astrocytes_Input_L002_R1.fastq.gz
ChIPseq_C9ALS_A11_Astrocytes_Input_L002_R2.fastq.gz
ChIPseq_C9ALS_A11_Microglia_H3K27ac_L001_R1.fastq.gz
ChIPseq_C9ALS_A11_Microglia_H3K27ac_L001_R2.fastq.gz
ChIPseq_C9ALS_A11_Microglia_H3K27ac_L004_R1.fastq.gz
ChIPseq_C9ALS_A11_Microglia_H3K27ac_L004_R2.fastq.gz
ChIPseq_C9ALS_A11_Microglia_Input_L002_R1.fastq.gz
ChIPseq_C9ALS_A11_Microglia_Input_L002_R2.fastq.gz
ChIPseq_C9ALS_A11_Neurons_H3K27ac_L001_R1.fastq.gz
ChIPseq_C9ALS_A11_Neurons_H3K27ac_L001_R2.fastq.gz
ChIPseq_C9ALS_A11_Neurons_H3K27ac_L004_R1.fastq.gz
ChIPseq_C9ALS_A11_Neurons_H3K27ac_L004_R2.fastq.gz
ChIPseq_C9ALS_A11_Neurons_Input_L002_R1.fastq.gz
ChIPseq_C9ALS_A11_Neurons_Input_L002_R2.fastq.gz
ChIPseq_C9ALS_A11_OligoLineage_H3K27ac_L001_R1.fastq.gz
ChIPseq_C9ALS_A11_OligoLineage_H3K27ac_L001_R2.fastq.gz
ChIPseq_C9ALS_A11_OligoLineage_H3K27ac_L004_R1.fastq.gz
ChIPseq_C9ALS_A11_OligoLineage_H3K27ac_L004_R2.fastq.gz
ChIPseq_C9ALS_A11_OligoLineage_Input_L002_R1.fastq.gz
ChIPseq_C9ALS_A11_OligoLineage_Input_L002_R2.fastq.gz
ChIPseq_C9ALS_A3_Astrocytes_H3K27ac_L001_R1.fastq.gz
ChIPseq_C9ALS_A3_Astrocytes_H3K27ac_L001_R2.fastq.gz
ChIPseq_C9ALS_A3_Astrocytes_H3K27ac_L004_R1.fastq.gz
ChIPseq_C9ALS_A3_Astrocytes_H3K27ac_L004_R2.fastq.gz
ChIPseq_C9ALS_A3_Astrocytes_Input_L002_R1.fastq.gz
ChIPseq_C9ALS_A3_Astrocytes_Input_L002_R2.fastq.gz
ChIPseq_C9ALS_A3_Microglia_H3K27ac_L001_R1.fastq.gz
ChIPseq_C9ALS_A3_Microglia_H3K27ac_L001_R2.fastq.gz
ChIPseq_C9ALS_A3_Microglia_H3K27ac_L004_R1.fastq.gz
ChIPseq_C9ALS_A3_Microglia_H3K27ac_L004_R2.fastq.gz
ChIPseq_C9ALS_A3_Microglia_Input_L002_R1.fastq.gz
ChIPseq_C9ALS_A3_Microglia_Input_L002_R2.fastq.gz
ChIPseq_C9ALS_A3_Neurons_H3K27ac_L001_R1.fastq.gz
ChIPseq_C9ALS_A3_Neurons_H3K27ac_L001_R2.fastq.gz
ChIPseq_C9ALS_A3_Neurons_H3K27ac_L004_R1.fastq.gz
ChIPseq_C9ALS_A3_Neurons_H3K27ac_L004_R2.fastq.gz
ChIPseq_C9ALS_A3_Neurons_Input_L002_R1.fastq.gz
ChIPseq_C9ALS_A3_Neurons_Input_L002_R2.fastq.gz
ChIPseq_C9ALS_A3_OligoLineage_H3K27ac_L001_R1.fastq.gz
ChIPseq_C9ALS_A3_OligoLineage_H3K27ac_L001_R2.fastq.gz
ChIPseq_C9ALS_A3_OligoLineage_H3K27ac_L004_R1.fastq.gz
ChIPseq_C9ALS_A3_OligoLineage_H3K27ac_L004_R2.fastq.gz
ChIPseq_C9ALS_A3_OligoLineage_Input_L002_R1.fastq.gz
ChIPseq_C9ALS_A3_OligoLineage_Input_L002_R2.fastq.gz
ChIPseq_C9ALS_A7_Astrocytes_H3K27ac_L001_R1.fastq.gz
ChIPseq_C9ALS_A7_Astrocytes_H3K27ac_L001_R2.fastq.gz
ChIPseq_C9ALS_A7_Astrocytes_H3K27ac_L004_R1.fastq.gz
ChIPseq_C9ALS_A7_Astrocytes_H3K27ac_L004_R2.fastq.gz
ChIPseq_C9ALS_A7_Microglia_H3K27ac_L001_R1.fastq.gz
ChIPseq_C9ALS_A7_Microglia_H3K27ac_L001_R2.fastq.gz
ChIPseq_C9ALS_A7_Microglia_H3K27ac_L004_R1.fastq.gz
ChIPseq_C9ALS_A7_Microglia_H3K27ac_L004_R2.fastq.gz

ChIPseq_C9ALS_A7_Neurons_H3K27ac_L001_R1.fastq.gz
ChIPseq_C9ALS_A7_Neurons_H3K27ac_L001_R2.fastq.gz
ChIPseq_C9ALS_A7_Neurons_H3K27ac_L004_R1.fastq.gz
ChIPseq_C9ALS_A7_Neurons_H3K27ac_L004_R2.fastq.gz
ChIPseq_C9ALS_A7_OligoLineage_H3K27ac_L001_R1.fastq.gz
ChIPseq_C9ALS_A7_OligoLineage_H3K27ac_L001_R2.fastq.gz
ChIPseq_C9ALS_A7_OligoLineage_H3K27ac_L004_R1.fastq.gz
ChIPseq_C9ALS_A7_OligoLineage_H3K27ac_L004_R2.fastq.gz
ChIPseq_C9ALS_A8_Astrocytes_H3K27ac_L001_R1.fastq.gz
ChIPseq_C9ALS_A8_Astrocytes_H3K27ac_L001_R2.fastq.gz
ChIPseq_C9ALS_A8_Astrocytes_H3K27ac_L004_R1.fastq.gz
ChIPseq_C9ALS_A8_Astrocytes_H3K27ac_L004_R2.fastq.gz
ChIPseq_C9ALS_A8_Microglia_H3K27ac_L001_R1.fastq.gz
ChIPseq_C9ALS_A8_Microglia_H3K27ac_L001_R2.fastq.gz
ChIPseq_C9ALS_A8_Microglia_H3K27ac_L004_R1.fastq.gz
ChIPseq_C9ALS_A8_Microglia_H3K27ac_L004_R2.fastq.gz
ChIPseq_C9ALS_A8_Neurons_H3K27ac_L001_R1.fastq.gz
ChIPseq_C9ALS_A8_Neurons_H3K27ac_L001_R2.fastq.gz
ChIPseq_C9ALS_A8_Neurons_H3K27ac_L004_R1.fastq.gz
ChIPseq_C9ALS_A8_Neurons_H3K27ac_L004_R2.fastq.gz
ChIPseq_C9ALS_A8_OligoLineage_H3K27ac_L001_R1.fastq.gz
ChIPseq_C9ALS_A8_OligoLineage_H3K27ac_L001_R2.fastq.gz
ChIPseq_C9ALS_A8_OligoLineage_H3K27ac_L004_R1.fastq.gz
ChIPseq_C9ALS_A8_OligoLineage_H3K27ac_L004_R2.fastq.gz
ChIPseq_C9ALS_A9_Astrocytes_H3K27ac_L001_R1.fastq.gz
ChIPseq_C9ALS_A9_Astrocytes_H3K27ac_L001_R2.fastq.gz
ChIPseq_C9ALS_A9_Astrocytes_H3K27ac_L004_R1.fastq.gz
ChIPseq_C9ALS_A9_Astrocytes_H3K27ac_L004_R2.fastq.gz
ChIPseq_C9ALS_A9_Microglia_H3K27ac_L001_R1.fastq.gz
ChIPseq_C9ALS_A9_Microglia_H3K27ac_L001_R2.fastq.gz
ChIPseq_C9ALS_A9_Microglia_H3K27ac_L004_R1.fastq.gz
ChIPseq_C9ALS_A9_Microglia_H3K27ac_L004_R2.fastq.gz
ChIPseq_C9ALS_A9_Neurons_H3K27ac_L001_R1.fastq.gz
ChIPseq_C9ALS_A9_Neurons_H3K27ac_L001_R2.fastq.gz
ChIPseq_C9ALS_A9_Neurons_H3K27ac_L004_R1.fastq.gz
ChIPseq_C9ALS_A9_Neurons_H3K27ac_L004_R2.fastq.gz
ChIPseq_C9ALS_A9_OligoLineage_H3K27ac_L001_R1.fastq.gz
ChIPseq_C9ALS_A9_OligoLineage_H3K27ac_L001_R2.fastq.gz
ChIPseq_C9ALS_A9_OligoLineage_H3K27ac_L004_R1.fastq.gz
ChIPseq_C9ALS_A9_OligoLineage_H3K27ac_L004_R2.fastq.gz
ChIPseq_Control_C10_Astrocytes_H3K27ac_L001_R1.fastq.gz
ChIPseq_Control_C10_Astrocytes_H3K27ac_L001_R2.fastq.gz
ChIPseq_Control_C10_Astrocytes_H3K27ac_L004_R1.fastq.gz
ChIPseq_Control_C10_Astrocytes_H3K27ac_L004_R2.fastq.gz
ChIPseq_Control_C10_Microglia_H3K27ac_L001_R1.fastq.gz
ChIPseq_Control_C10_Microglia_H3K27ac_L001_R2.fastq.gz
ChIPseq_Control_C10_Microglia_H3K27ac_L004_R1.fastq.gz
ChIPseq_Control_C10_Microglia_H3K27ac_L004_R2.fastq.gz
ChIPseq_Control_C10_Neurons_H3K27ac_L001_R1.fastq.gz
ChIPseq_Control_C10_Neurons_H3K27ac_L001_R2.fastq.gz
ChIPseq_Control_C10_Neurons_H3K27ac_L004_R1.fastq.gz
ChIPseq_Control_C10_Neurons_H3K27ac_L004_R2.fastq.gz
ChIPseq_Control_C10_OligoLineage_H3K27ac_L001_R1.fastq.gz
ChIPseq_Control_C10_OligoLineage_H3K27ac_L001_R2.fastq.gz
ChIPseq_Control_C10_OligoLineage_H3K27ac_L004_R1.fastq.gz
ChIPseq_Control_C10_OligoLineage_H3K27ac_L004_R2.fastq.gz
ChIPseq_Control_C11_Astrocytes_H3K27ac_L001_R1.fastq.gz
ChIPseq_Control_C11_Astrocytes_H3K27ac_L001_R2.fastq.gz
ChIPseq_Control_C11_Astrocytes_H3K27ac_L004_R1.fastq.gz
ChIPseq_Control_C11_Astrocytes_H3K27ac_L004_R2.fastq.gz
ChIPseq_Control_C11_Microglia_H3K27ac_L001_R1.fastq.gz
ChIPseq_Control_C11_Microglia_H3K27ac_L001_R2.fastq.gz
ChIPseq_Control_C11_Microglia_H3K27ac_L004_R1.fastq.gz
ChIPseq_Control_C11_Microglia_H3K27ac_L004_R2.fastq.gz
ChIPseq_Control_C11_Neurons_H3K27ac_L001_R1.fastq.gz
ChIPseq_Control_C11_Neurons_H3K27ac_L001_R2.fastq.gz
ChIPseq_Control_C11_Neurons_H3K27ac_L004_R1.fastq.gz
ChIPseq_Control_C11_Neurons_H3K27ac_L004_R2.fastq.gz
ChIPseq_Control_C11_OligoLineage_H3K27ac_L001_R1.fastq.gz
ChIPseq_Control_C11_OligoLineage_H3K27ac_L001_R2.fastq.gz
ChIPseq_Control_C11_OligoLineage_H3K27ac_L004_R1.fastq.gz
ChIPseq_Control_C11_OligoLineage_H3K27ac_L004_R2.fastq.gz
ChIPseq_Control_C12_Astrocytes_H3K27ac_L001_R1.fastq.gz
ChIPseq_Control_C12_Astrocytes_H3K27ac_L001_R2.fastq.gz
ChIPseq_Control_C12_Astrocytes_H3K27ac_L004_R1.fastq.gz
ChIPseq_Control_C12_Astrocytes_H3K27ac_L004_R2.fastq.gz

ChIPseq_Control_C12_Microglia_H3K27ac_L001_R1.fastq.gz
ChIPseq_Control_C12_Microglia_H3K27ac_L001_R2.fastq.gz
ChIPseq_Control_C12_Microglia_H3K27ac_L004_R1.fastq.gz
ChIPseq_Control_C12_Microglia_H3K27ac_L004_R2.fastq.gz
ChIPseq_Control_C12_Neurons_H3K27ac_L001_R1.fastq.gz
ChIPseq_Control_C12_Neurons_H3K27ac_L001_R2.fastq.gz
ChIPseq_Control_C12_Neurons_H3K27ac_L004_R1.fastq.gz
ChIPseq_Control_C12_Neurons_H3K27ac_L004_R2.fastq.gz
ChIPseq_Control_C12_OligoLineage_H3K27ac_L001_R1.fastq.gz
ChIPseq_Control_C12_OligoLineage_H3K27ac_L001_R2.fastq.gz
ChIPseq_Control_C12_OligoLineage_H3K27ac_L004_R1.fastq.gz
ChIPseq_Control_C12_OligoLineage_H3K27ac_L004_R2.fastq.gz
ChIPseq_Control_C13_Astrocytes_H3K27ac_L001_R1.fastq.gz
ChIPseq_Control_C13_Astrocytes_H3K27ac_L001_R2.fastq.gz
ChIPseq_Control_C13_Astrocytes_H3K27ac_L004_R1.fastq.gz
ChIPseq_Control_C13_Astrocytes_H3K27ac_L004_R2.fastq.gz
ChIPseq_Control_C13_Microglia_H3K27ac_L001_R1.fastq.gz
ChIPseq_Control_C13_Microglia_H3K27ac_L001_R2.fastq.gz
ChIPseq_Control_C13_Microglia_H3K27ac_L004_R1.fastq.gz
ChIPseq_Control_C13_Microglia_H3K27ac_L004_R2.fastq.gz
ChIPseq_Control_C13_Neurons_H3K27ac_L001_R1.fastq.gz
ChIPseq_Control_C13_Neurons_H3K27ac_L001_R2.fastq.gz
ChIPseq_Control_C13_Neurons_H3K27ac_L004_R1.fastq.gz
ChIPseq_Control_C13_Neurons_H3K27ac_L004_R2.fastq.gz
ChIPseq_Control_C14_Astrocytes_H3K27ac_L001_R1.fastq.gz
ChIPseq_Control_C14_Astrocytes_H3K27ac_L001_R2.fastq.gz
ChIPseq_Control_C14_Astrocytes_H3K27ac_L004_R1.fastq.gz
ChIPseq_Control_C14_Astrocytes_H3K27ac_L004_R2.fastq.gz
ChIPseq_Control_C14_Astrocytes_Input_L002_R1.fastq.gz
ChIPseq_Control_C14_Astrocytes_Input_L002_R2.fastq.gz
ChIPseq_Control_C14_Microglia_H3K27ac_L001_R1.fastq.gz
ChIPseq_Control_C14_Microglia_H3K27ac_L001_R2.fastq.gz
ChIPseq_Control_C14_Microglia_H3K27ac_L004_R1.fastq.gz
ChIPseq_Control_C14_Microglia_H3K27ac_L004_R2.fastq.gz
ChIPseq_Control_C14_Microglia_Input_L002_R1.fastq.gz
ChIPseq_Control_C14_Microglia_Input_L002_R2.fastq.gz
ChIPseq_Control_C14_Neurons_H3K27ac_L001_R1.fastq.gz
ChIPseq_Control_C14_Neurons_H3K27ac_L001_R2.fastq.gz
ChIPseq_Control_C14_Neurons_H3K27ac_L004_R1.fastq.gz
ChIPseq_Control_C14_Neurons_H3K27ac_L004_R2.fastq.gz
ChIPseq_Control_C14_Neurons_Input_L002_R1.fastq.gz
ChIPseq_Control_C14_Neurons_Input_L002_R2.fastq.gz
ChIPseq_Control_C14_OligoLineage_H3K27ac_L001_R1.fastq.gz
ChIPseq_Control_C14_OligoLineage_H3K27ac_L001_R2.fastq.gz
ChIPseq_Control_C14_OligoLineage_H3K27ac_L004_R1.fastq.gz
ChIPseq_Control_C14_OligoLineage_H3K27ac_L004_R2.fastq.gz
ChIPseq_Control_C14_OligoLineage_Input_L002_R1.fastq.gz
ChIPseq_Control_C14_OligoLineage_Input_L002_R2.fastq.gz
ChIPseq_Control_C15_Astrocytes_H3K27ac_L001_R1.fastq.gz
ChIPseq_Control_C15_Astrocytes_H3K27ac_L001_R2.fastq.gz
ChIPseq_Control_C15_Astrocytes_H3K27ac_L004_R1.fastq.gz
ChIPseq_Control_C15_Astrocytes_H3K27ac_L004_R2.fastq.gz
ChIPseq_Control_C15_Microglia_H3K27ac_L001_R1.fastq.gz
ChIPseq_Control_C15_Microglia_H3K27ac_L001_R2.fastq.gz
ChIPseq_Control_C15_Microglia_H3K27ac_L004_R1.fastq.gz
ChIPseq_Control_C15_Microglia_H3K27ac_L004_R2.fastq.gz
ChIPseq_Control_C15_Neurons_H3K27ac_L001_R1.fastq.gz
ChIPseq_Control_C15_Neurons_H3K27ac_L001_R2.fastq.gz
ChIPseq_Control_C15_Neurons_H3K27ac_L004_R1.fastq.gz
ChIPseq_Control_C15_Neurons_H3K27ac_L004_R2.fastq.gz
ChIPseq_Control_C15_OligoLineage_H3K27ac_L001_R1.fastq.gz
ChIPseq_Control_C15_OligoLineage_H3K27ac_L001_R2.fastq.gz
ChIPseq_Control_C15_OligoLineage_H3K27ac_L004_R1.fastq.gz
ChIPseq_Control_C15_OligoLineage_H3K27ac_L004_R2.fastq.gz
ChIPseq_Control_C7_Astrocytes_H3K27ac_L001_R1.fastq.gz
ChIPseq_Control_C7_Astrocytes_H3K27ac_L001_R2.fastq.gz
ChIPseq_Control_C7_Astrocytes_H3K27ac_L004_R1.fastq.gz
ChIPseq_Control_C7_Astrocytes_H3K27ac_L004_R2.fastq.gz
ChIPseq_Control_C7_Astrocytes_Input_L002_R1.fastq.gz
ChIPseq_Control_C7_Astrocytes_Input_L002_R2.fastq.gz
ChIPseq_Control_C7_Microglia_H3K27ac_L001_R1.fastq.gz
ChIPseq_Control_C7_Microglia_H3K27ac_L001_R2.fastq.gz
ChIPseq_Control_C7_Microglia_H3K27ac_L004_R1.fastq.gz
ChIPseq_Control_C7_Microglia_H3K27ac_L004_R2.fastq.gz
ChIPseq_Control_C7_Microglia_Input_L002_R1.fastq.gz
ChIPseq_Control_C7_Microglia_Input_L002_R2.fastq.gz

ChIPseq_Control_C7_Neurons_H3K27ac_L001_R1.fastq.gz
ChIPseq_Control_C7_Neurons_H3K27ac_L001_R2.fastq.gz
ChIPseq_Control_C7_Neurons_H3K27ac_L004_R1.fastq.gz
ChIPseq_Control_C7_Neurons_H3K27ac_L004_R2.fastq.gz
ChIPseq_Control_C7_Neurons_Input_L002_R1.fastq.gz
ChIPseq_Control_C7_Neurons_Input_L002_R2.fastq.gz
ChIPseq_Control_C7_OligoLineage_H3K27ac_L001_R1.fastq.gz
ChIPseq_Control_C7_OligoLineage_H3K27ac_L001_R2.fastq.gz
ChIPseq_Control_C7_OligoLineage_H3K27ac_L004_R1.fastq.gz
ChIPseq_Control_C7_OligoLineage_H3K27ac_L004_R2.fastq.gz
ChIPseq_Control_C7_OligoLineage_Input_L002_R1.fastq.gz
ChIPseq_Control_C7_OligoLineage_Input_L002_R2.fastq.gz
ChIPseq_Control_C8_Astrocytes_H3K27ac_L001_R1.fastq.gz
ChIPseq_Control_C8_Astrocytes_H3K27ac_L001_R2.fastq.gz
ChIPseq_Control_C8_Astrocytes_H3K27ac_L004_R1.fastq.gz
ChIPseq_Control_C8_Astrocytes_H3K27ac_L004_R2.fastq.gz
ChIPseq_Control_C8_Microglia_H3K27ac_L001_R1.fastq.gz
ChIPseq_Control_C8_Microglia_H3K27ac_L001_R2.fastq.gz
ChIPseq_Control_C8_Microglia_H3K27ac_L004_R1.fastq.gz
ChIPseq_Control_C8_Microglia_H3K27ac_L004_R2.fastq.gz
ChIPseq_Control_C8_Neurons_H3K27ac_L001_R1.fastq.gz
ChIPseq_Control_C8_Neurons_H3K27ac_L001_R2.fastq.gz
ChIPseq_Control_C8_Neurons_H3K27ac_L004_R1.fastq.gz
ChIPseq_Control_C8_Neurons_H3K27ac_L004_R2.fastq.gz
ChIPseq_Control_C8_OligoLineage_H3K27ac_L001_R1.fastq.gz
ChIPseq_Control_C8_OligoLineage_H3K27ac_L001_R2.fastq.gz
ChIPseq_Control_C8_OligoLineage_H3K27ac_L004_R1.fastq.gz
ChIPseq_Control_C8_OligoLineage_H3K27ac_L004_R2.fastq.gz
ChIPseq_Control_C9_Astrocytes_H3K27ac_L001_R1.fastq.gz
ChIPseq_Control_C9_Astrocytes_H3K27ac_L001_R2.fastq.gz
ChIPseq_Control_C9_Astrocytes_H3K27ac_L004_R1.fastq.gz
ChIPseq_Control_C9_Astrocytes_H3K27ac_L004_R2.fastq.gz
ChIPseq_Control_C9_Astrocytes_Input_L001_R1.fastq.gz
ChIPseq_Control_C9_Astrocytes_Input_L001_R2.fastq.gz
ChIPseq_Control_C9_Astrocytes_Input_L004_R1.fastq.gz
ChIPseq_Control_C9_Astrocytes_Input_L004_R2.fastq.gz
ChIPseq_Control_C9_Microglia_H3K27ac_L001_R1.fastq.gz
ChIPseq_Control_C9_Microglia_H3K27ac_L001_R2.fastq.gz
ChIPseq_Control_C9_Microglia_H3K27ac_L004_R1.fastq.gz
ChIPseq_Control_C9_Microglia_H3K27ac_L004_R2.fastq.gz
ChIPseq_Control_C9_Microglia_Input_L001_R1.fastq.gz
ChIPseq_Control_C9_Microglia_Input_L001_R2.fastq.gz
ChIPseq_Control_C9_Microglia_Input_L004_R1.fastq.gz
ChIPseq_Control_C9_Microglia_Input_L004_R2.fastq.gz
ChIPseq_Control_C9_Neurons_H3K27ac_L001_R1.fastq.gz
ChIPseq_Control_C9_Neurons_H3K27ac_L001_R2.fastq.gz
ChIPseq_Control_C9_Neurons_H3K27ac_L004_R1.fastq.gz
ChIPseq_Control_C9_Neurons_H3K27ac_L004_R2.fastq.gz
ChIPseq_Control_C9_Neurons_Input_L001_R1.fastq.gz
ChIPseq_Control_C9_Neurons_Input_L001_R2.fastq.gz
ChIPseq_Control_C9_Neurons_Input_L004_R1.fastq.gz
ChIPseq_Control_C9_Neurons_Input_L004_R2.fastq.gz
ChIPseq_Control_C9_OligoLineage_H3K27ac_L001_R1.fastq.gz
ChIPseq_Control_C9_OligoLineage_H3K27ac_L001_R2.fastq.gz
ChIPseq_Control_C9_OligoLineage_H3K27ac_L004_R1.fastq.gz
ChIPseq_Control_C9_OligoLineage_H3K27ac_L004_R2.fastq.gz
ChIPseq_Control_C9_OligoLineage_Input_L001_R1.fastq.gz
ChIPseq_Control_C9_OligoLineage_Input_L001_R2.fastq.gz
ChIPseq_Control_C9_OligoLineage_Input_L004_R1.fastq.gz
ChIPseq_Control_C9_OligoLineage_Input_L004_R2.fastq.gz
ChIPseq_C9ALS_A10_Astrocytes_H3K27ac_broadPeak.tsv
ChIPseq_C9ALS_A10_Microglia_H3K27ac_broadPeak.tsv
ChIPseq_C9ALS_A10_Neurons_H3K27ac_broadPeak.tsv
ChIPseq_C9ALS_A10_OligoLineage_H3K27ac_broadPeak.tsv
ChIPseq_C9ALS_A11_Astrocytes_H3K27ac_broadPeak.tsv
ChIPseq_C9ALS_A11_Microglia_H3K27ac_broadPeak.tsv
ChIPseq_C9ALS_A11_Neurons_H3K27ac_broadPeak.tsv
ChIPseq_C9ALS_A11_OligoLineage_H3K27ac_broadPeak.tsv
ChIPseq_C9ALS_A3_Astrocytes_H3K27ac_broadPeak.tsv
ChIPseq_C9ALS_A3_Microglia_H3K27ac_broadPeak.tsv
ChIPseq_C9ALS_A3_Neurons_H3K27ac_broadPeak.tsv
ChIPseq_C9ALS_A3_OligoLineage_H3K27ac_broadPeak.tsv
ChIPseq_C9ALS_A7_Astrocytes_H3K27ac_broadPeak.tsv
ChIPseq_C9ALS_A7_Microglia_H3K27ac_broadPeak.tsv
ChIPseq_C9ALS_A7_Neurons_H3K27ac_broadPeak.tsv
ChIPseq_C9ALS_A7_OligoLineage_H3K27ac_broadPeak.tsv

ChIPseq_C9ALS_A8_Astrocytes_H3K27ac_broadPeak.tsv
ChIPseq_C9ALS_A8_Microglia_H3K27ac_broadPeak.tsv
ChIPseq_C9ALS_A8_Neurons_H3K27ac_broadPeak.tsv
ChIPseq_C9ALS_A8_OligoLineage_H3K27ac_broadPeak.tsv
ChIPseq_C9ALS_A9_Astrocytes_H3K27ac_broadPeak.tsv
ChIPseq_C9ALS_A9_Microglia_H3K27ac_broadPeak.tsv
ChIPseq_C9ALS_A9_Neurons_H3K27ac_broadPeak.tsv
ChIPseq_C9ALS_A9_OligoLineage_H3K27ac_broadPeak.tsv
ChIPseq_Control_C10_Astrocytes_H3K27ac_broadPeak.tsv
ChIPseq_Control_C10_Microglia_H3K27ac_broadPeak.tsv
ChIPseq_Control_C10_Neurons_H3K27ac_broadPeak.tsv
ChIPseq_Control_C10_OligoLineage_H3K27ac_broadPeak.tsv
ChIPseq_Control_C11_Astrocytes_H3K27ac_broadPeak.tsv
ChIPseq_Control_C11_Microglia_H3K27ac_broadPeak.tsv
ChIPseq_Control_C11_Neurons_H3K27ac_broadPeak.tsv
ChIPseq_Control_C11_OligoLineage_H3K27ac_broadPeak.tsv
ChIPseq_Control_C12_Astrocytes_H3K27ac_broadPeak.tsv
ChIPseq_Control_C12_Microglia_H3K27ac_broadPeak.tsv
ChIPseq_Control_C12_Neurons_H3K27ac_broadPeak.tsv
ChIPseq_Control_C12_OligoLineage_H3K27ac_broadPeak.tsv
ChIPseq_Control_C13_Astrocytes_H3K27ac_broadPeak.tsv
ChIPseq_Control_C13_Microglia_H3K27ac_broadPeak.tsv
ChIPseq_Control_C13_Neurons_H3K27ac_broadPeak.tsv
ChIPseq_Control_C14_Astrocytes_H3K27ac_broadPeak.tsv
ChIPseq_Control_C14_Microglia_H3K27ac_broadPeak.tsv
ChIPseq_Control_C14_Neurons_H3K27ac_broadPeak.tsv
ChIPseq_Control_C14_OligoLineage_H3K27ac_broadPeak.tsv
ChIPseq_Control_C15_Astrocytes_H3K27ac_broadPeak.tsv
ChIPseq_Control_C15_Microglia_H3K27ac_broadPeak.tsv
ChIPseq_Control_C15_Neurons_H3K27ac_broadPeak.tsv
ChIPseq_Control_C15_OligoLineage_H3K27ac_broadPeak.tsv
ChIPseq_Control_C7_Astrocytes_H3K27ac_broadPeak.tsv
ChIPseq_Control_C7_Microglia_H3K27ac_broadPeak.tsv
ChIPseq_Control_C7_Neurons_H3K27ac_broadPeak.tsv
ChIPseq_Control_C7_OligoLineage_H3K27ac_broadPeak.tsv
ChIPseq_Control_C8_Astrocytes_H3K27ac_broadPeak.tsv
ChIPseq_Control_C8_Microglia_H3K27ac_broadPeak.tsv
ChIPseq_Control_C8_Neurons_H3K27ac_broadPeak.tsv
ChIPseq_Control_C8_OligoLineage_H3K27ac_broadPeak.tsv
ChIPseq_Control_C9_Astrocytes_H3K27ac_broadPeak.tsv
ChIPseq_Control_C9_Microglia_H3K27ac_broadPeak.tsv
ChIPseq_Control_C9_Neurons_H3K27ac_broadPeak.tsv
ChIPseq_Control_C9_OligoLineage_H3K27ac_broadPeak.tsv
ChIPseq_C9ALS_A10_Astrocytes_H3K27ac_1M.bw
ChIPseq_C9ALS_A10_Microglia_H3K27ac_1M.bw
ChIPseq_C9ALS_A10_Neurons_H3K27ac_1M.bw
ChIPseq_C9ALS_A10_OligoLineage_H3K27ac_1M.bw
ChIPseq_C9ALS_A11_Astrocytes_H3K27ac_1M.bw
ChIPseq_C9ALS_A11_Microglia_H3K27ac_1M.bw
ChIPseq_C9ALS_A11_Neurons_H3K27ac_1M.bw
ChIPseq_C9ALS_A11_OligoLineage_H3K27ac_1M.bw
ChIPseq_C9ALS_A3_Astrocytes_H3K27ac_1M.bw
ChIPseq_C9ALS_A3_Microglia_H3K27ac_1M.bw
ChIPseq_C9ALS_A3_Neurons_H3K27ac_1M.bw
ChIPseq_C9ALS_A3_OligoLineage_H3K27ac_1M.bw
ChIPseq_C9ALS_A7_Astrocytes_H3K27ac_1M.bw
ChIPseq_C9ALS_A7_Microglia_H3K27ac_1M.bw
ChIPseq_C9ALS_A7_Neurons_H3K27ac_1M.bw
ChIPseq_C9ALS_A7_OligoLineage_H3K27ac_1M.bw
ChIPseq_C9ALS_A8_Astrocytes_H3K27ac_1M.bw
ChIPseq_C9ALS_A8_Microglia_H3K27ac_1M.bw
ChIPseq_C9ALS_A8_Neurons_H3K27ac_1M.bw
ChIPseq_C9ALS_A8_OligoLineage_H3K27ac_1M.bw
ChIPseq_C9ALS_A9_Astrocytes_H3K27ac_1M.bw
ChIPseq_C9ALS_A9_Microglia_H3K27ac_1M.bw
ChIPseq_C9ALS_A9_Neurons_H3K27ac_1M.bw
ChIPseq_C9ALS_A9_OligoLineage_H3K27ac_1M.bw
ChIPseq_Control_C10_Astrocytes_H3K27ac_1M.bw
ChIPseq_Control_C10_Microglia_H3K27ac_1M.bw
ChIPseq_Control_C10_Neurons_H3K27ac_1M.bw
ChIPseq_Control_C10_OligoLineage_H3K27ac_1M.bw
ChIPseq_Control_C11_Astrocytes_H3K27ac_1M.bw
ChIPseq_Control_C11_Microglia_H3K27ac_1M.bw
ChIPseq_Control_C11_Neurons_H3K27ac_1M.bw
ChIPseq_Control_C11_OligoLineage_H3K27ac_1M.bw
ChIPseq_Control_C12_Astrocytes_H3K27ac_1M.bw

ChIPseq_Control_C12_Microglia_H3K27ac_1M.bw
ChIPseq_Control_C12_Neurons_H3K27ac_1M.bw
ChIPseq_Control_C12_OligoLineage_H3K27ac_1M.bw
ChIPseq_Control_C13_Astrocytes_H3K27ac_1M.bw
ChIPseq_Control_C13_Microglia_H3K27ac_1M.bw
ChIPseq_Control_C13_Neurons_H3K27ac_1M.bw
ChIPseq_Control_C14_Astrocytes_H3K27ac_1M.bw
ChIPseq_Control_C14_Microglia_H3K27ac_1M.bw
ChIPseq_Control_C14_Neurons_H3K27ac_1M.bw
ChIPseq_Control_C14_OligoLineage_H3K27ac_1M.bw
ChIPseq_Control_C15_Astrocytes_H3K27ac_1M.bw
ChIPseq_Control_C15_Microglia_H3K27ac_1M.bw
ChIPseq_Control_C15_Neurons_H3K27ac_1M.bw
ChIPseq_Control_C15_OligoLineage_H3K27ac_1M.bw
ChIPseq_Control_C7_Astrocytes_H3K27ac_1M.bw
ChIPseq_Control_C7_Microglia_H3K27ac_1M.bw
ChIPseq_Control_C7_Neurons_H3K27ac_1M.bw
ChIPseq_Control_C7_OligoLineage_H3K27ac_1M.bw
ChIPseq_Control_C8_Astrocytes_H3K27ac_1M.bw
ChIPseq_Control_C8_Microglia_H3K27ac_1M.bw
ChIPseq_Control_C8_Neurons_H3K27ac_1M.bw
ChIPseq_Control_C8_OligoLineage_H3K27ac_1M.bw
ChIPseq_Control_C9_Astrocytes_H3K27ac_1M.bw
ChIPseq_Control_C9_Microglia_H3K27ac_1M.bw
ChIPseq_Control_C9_Neurons_H3K27ac_1M.bw
ChIPseq_Control_C9_OligoLineage_H3K27ac_1M.bw

Genome browser session
(e.g. UCSC)

https://brainome.ucsd.edu/C9_ALS_FTD/

Methodology

Replicates

Biological replicates for ChIP-seq experiments were from N=9 control subjects and N=6 C9orf72(+) ALS subjects. There was high agreement between samples of the same cell type, as validated by high correlation of H3K27ac signal within each cell type (Fig. 5B).

Sequencing depth

Sequencing was performed with a paired-end 100-cycle protocol to ~60 million reads per library. Raw data (FASTQ files) were pre-processed to remove adapters, low quality sequences and PCR duplicates using the HTStream software tool and mapped to the hg38 genome build with the BWA MEM software (github.com/bwa-mem2) using default parameters (~94% uniquely mapped reads).

Antibodies

Anti-H3K27ac antibodies were from ActiveMotif, (#39133), validated for H3K27ac ChIP-seq by the company, and in multiple publications from ourselves and other groups.

Peak calling parameters

H3K27ac peaks were called using MACS2 [pypi.org/project/MACS2, (Feng, Liu, Qin, Zhang, & Liu, 2012)] including input controls for each cell type and age group and the following command line settings: callpeak -B -f BAMPE --broad --broad-cutoff 0.00001, as described previously (see Methods for more details).

Data quality

ChIP-seq data were collected and analyzed using previously established protocols (Kozlenkov et al, 2020) and were similar in mapping rates, signal-to-noise ratio and numbers of called peaks to the previously published results. Based on our analyses (Fig. 5B), the outputs of BWA and MACS2 software, and the inspection of the bigwig traces (e.g., Fig. 5C), the data had high signal-to-noise ratio and a high agreement between replicates of the same cell type and diagnosis.

Software

ChIP-seq data were pre-processed with HTStream, mapped with BWA MEM, and peaks were called with MACS2. See more details in the Methods section.

Flow Cytometry

Plots

Confirm that:

☒ The axis labels state the marker and fluorochrome used (e.g. CD4-FITC).

☒ The axis scales are clearly visible. Include numbers along axes only for bottom left plot of group (a 'group' is an analysis of identical markers).

☒ All plots are contour plots with outliers or pseudocolor plots.

☒ A numerical value for number of cells or percentage (with statistics) is provided.

Methodology

Sample preparation

Fluorescence-activated nuclei sorting (FANS) and nuclear RNA-seq of the three populations of the human brain cells (neurons, oligodendrocyte lineage cells, and other glia)
Tissue (~300 mg) was homogenized in ice-cold lysis buffer (320 mM sucrose, 5 mM CaCl2, 3 mM Mg(Ac)2, 0.1 mM EDTA, 0.1% Triton X-100, 1 mM DTT, 1 U/µl RRI, and 10mM Tris-HCl, pH 8.0), underlaid with the sucrose buffer (1.8 M Sucrose, 3 mM Mg(Ac)2, 1 mM DTT, 0.4 U/µl RRI, and 10 mM Tris-HCl pH 8.0), and centrifuged for 1h at 98,600 g (24,000 rpm using

SW41Ti rotor). The nuclear pellets were then resuspended in the antibody-incubation buffer (0.5% BSA, 3mM MgCl2, 1 U/µl RRI, and 10 mM Tris-HCl, pH 8.0) and incubated with antibodies against NeuN and SOX10 for 1 hr at 4°C. FANS method was then used to isolate neurons (NeuN+SOX10- population), oligodendrocyte lineage cells (NeuN-SOX10+ population), consisting of mature oligodendrocytes and a smaller population of OPCs, and a third population (NeuN-SOX10-; "other glia") that mostly consisted of astrocytes and microglia.

We used Alexa488-conjugated anti-NeuN antibodies (1:1000 dilution, Millipore, Cat. # MAB377x) and anti-SOX10 antibodies (R&D Systems, Cat. # AF2864) that were custom conjugated to Alexa647 (1:150 dilution, Cat# FCMAB317PE, Millipore). DNA stain DAPI was used to label intact nuclei (see Supplementary Fig. 5 for details). From each sample, we collected 250-300 thousand (K) nuclei of neurons, 250-300K of oligodendrocyte lineage cells, and 150-200K nuclei of other glia. Nuclei were collected directly to the lysis buffer from PicoPure RNA Isolation Kit (ThermoFisher Scientific, Cat. # KIT024), which was then used for RNA isolation. RNA-seq libraries were prepared with the SMARTer Stranded Total RNA-seq Kit, Pico-Input v2 (Takara, Cat. # 634414) from 10ng of the RNA.

FANS and H3K27ac ChIP-seq of the four populations of the brain cells (neurons, oligodendrocyte lineage, astrocytes, and microglia)
FANS protocol to isolate neurons, oligodendrocyte lineage, astrocytes, and microglia was performed as described in the previous section, except removing RRI from all buffers, adding 0.1 mM benzamidine, 0.1 mM phenylmethylsulfonyl fluoride (PMSF) to the lysis buffer, and adding antibodies against interferon response factor 5 (IRF5). IRF5 is highly enriched in cells of myeloid origin, including microglia. The IRF5 antibodies allowed the separation of astrocytes and microglia within the other glia (NeuN-OLIG10-) population (see Supplementary Fig. 5 for details). In this FANS protocol, we used PE-conjugated anti-NeuN antibodies (1:1,000 dilution, Millipore, Cat. # FCMAB317PE), anti-SOX10 antibodies (R&D Systems, Cat. # AF2864), which were custom-conjugated to Alexa647 (1:150 dilution), and Alexa488-conjugated anti-IRF5 antibodies (1:200 dilution, R&D Systems, Cat. # IC4508G).

We employed the native ChIP protocol (N-ChIP) in which chromatin fragmentation is performed using micrococcal nuclease (MNase) without crosslinking proteins to DNA. 100-150K of each cell type were collected and used for each ChIP reaction. We used anti-H3K27ac antibodies from Active Motif (Cat# 39133; rabbit polyclonal, 3 µg per sample). ChIP-Seq libraries were prepared with the NEBNext Ultra II DNA Library Prep Kit for Illumina (New England Biolabs, Cat. # E7645). For each diagnostic group, three input control samples obtained from MNAse-digested DNA were prepared and sequenced.

Instrument

The flow cytometer instrument BD Influx, with four lasers (355nm, 488nm, 561nm and 640nm) and a 70-micron nozzle was used in all experiments.

Software

The software BD FACS Sortware was used to perform the flow cytometry experiments. The nature of the study did not require a use of a dedicated flow cytometry analysis software, since the aim of the flow cytometry step was to isolate the nuclei of the four major cell brain populations.

Cell population abundance

The four isolated cell populations had the following average abundances: neurons: 36% +/- 10%, oligodendrocytes: 53% +/- 11%, microglia: 4.1% +/- 1.3%, astrocytes: 7.1% +/- 2.5% (see Extended Data Fig.5). In the initial stages of the protocol optimization, back-sorting of the sorted populations was performed to validate the purity of the collected populations.

Gating strategy

The gating strategy included the following steps: (1) FSC/SSC gating to ensure debris removal, (2) FSC/Trigger Pulse Width gating to exclude doublets, (3) DNA dye (DAPI) signal gating to select for undamaged nuclei with a full chromosomal content, (4) and (5) gating based on the fluorophore-conjugated cell-type-specific antibodies: NeuN-PE, SOX10-AF647, and IRF5-AF488, to isolate the nuclei of the four major brain cell populations. See Methods for the Extended Data Fig.5, illustrating the gating steps (4) and (5).

☒ Tick this box to confirm that a figure exemplifying the gating strategy is provided in the Supplementary Information.
